# Materials‐Guided Gene‐Ionizable Lipid Nanoparticles to Reverse Iron‐Associated Immune Resistance in Renal Cancer

**DOI:** 10.1002/advs.202600078

**Published:** 2026-06-09

**Authors:** Xin Jin, Yulong Hong, Chengliang Yin, Wanyang Guo, Yaxuan Wang, Ruijiang Zeng, Ruilin Liu, Zexian Ding, Xinlin Liu, Shangqing Ren, Qiyang Liang, Yaohui Wang, Xu Zhang, João Conde, Yuan Li, Xin Ma, Liangyou Gu

**Affiliations:** ^1^ Department of Urology The Second Xiangya Hospital Central South University Changsha Hunan China; ^2^ Uro‐Oncology Institute of Central South University Changsha Hunan China; ^3^ Department of Biomedical Engineeringfaculty of Engineering University Malaya Kuala Lumpur Malaysia; ^4^ Department of Urology The First Affiliated Hospital of Harbin Medical University Harbin China; ^5^ Robotic Minimally Invasive Surgery Center School of Medicine Sichuan Provincial People's Hospital University of Electronic Science and Technology of China Chengdu China; ^6^ Department of Urology Chinese PLA General Hospital Beijing China; ^7^ Comprehensive Health Research Centre (CHRC) NOVA Medical School Faculdade de Ciências Médicas NMS|FCM Universidade NOVA De Lisboa Lisboa Portugal

**Keywords:** CD8+ T cells, FDX1 palmitoylation, Iron overload, lipid nanoparticles, renal cell carcinoma

## Abstract

Iron overload is a common metabolic disturbance in cancer and contributes to poor outcomes in renal cell carcinoma (RCC), yet its effects on the tumour immune microenvironment remain unclear. Here we identify a previously unrecognized immunosuppressive axis in which iron overload downregulates the palmitoyltransferase ZDHHC12 in CD8^+^ T cells, leading to impaired palmitoylation of the mitochondrial protein FDX1. This stabilizes FDX1 and drives cuproptosis, a recently described copper‐dependent cell death pathway, thereby compromising T cell effector function and diminishing responses to immune checkpoint blockade. To restore T cell activity, we engineered lipid nanoparticles (ZDHHC12‐LNPs) for the delivery of Zdhhc12. These nanoparticles exhibited optimal physicochemical properties, efficiently restored FDX1 palmitoylation, rescued CD8^+^ T cell function, and synergized with PD‐1 blockade in preclinical RCC models without inducing systemic toxicity. Our findings uncover the iron‐ZDHHC12‐FDX1 axis as a metabolic checkpoint of T cell immunity and demonstrate a nanotechnology‐based strategy to overcome iron‐driven immunosuppression, offering translational potential for patients with iron‐overloaded RCC.

## 1 Introduction

Cancer is recognized as a systemic and holistic disease, with changes in various nutrients and metabolites in the body affecting cancer progression in diverse ways [1]. Iron is an essential trace element in the human body, yet iron disorders affect billions of patients worldwide, including cancer patients [2]. Early large‐scale population studies showed that individuals with moderately elevated iron levels have an increased risk of cancer [3, 4]. Compared to other solid tumors, renal cell carcinoma (RCC) exhibits significantly higher iron accumulation scores [5]. Furthermore, Mendelian randomization (MR), which utilizes genetic variants as instrumental variables to effectively minimize confounding bias and enhance the reliability of causal inference, has become a primary method for large‐scale causal inference in epidemiology [6, 7]. An MR study by Yi Lu et al., encompassing 5,219 RCC cases, demonstrated a causal association between each standard deviation increase in serum iron concentration and an elevated risk of RCC (male: OR = 1.595, 95% CI = 1.310–1.758, p = 0.0238; female: OR = 1.484, 95% CI = 1.197–2.337, p = 0.0210) [8]. Thus, existing epidemiological evidence indicates a causal link between iron overload, increased serum iron concentration, and an elevated risk of RCC. Clinical data indicate that serum ferritin levels are associated with disease stage and prognosis: patients with stage III/IV disease exhibit higher ferritin levels than those with stage I/II, and patients with elevated ferritin have reduced 5‐year survival rates [9, 10]. Approximately three‐quarters of RCC patients harbor VHL mutations [11], which lead to abnormal accumulation of hypoxia‐inducible factors (HIFs), activating pro‐angiogenic signaling pathways such as vascular endothelial growth factor (VEGF) and promoting abnormal tumor angiogenesis [12, 13]. Studies have shown that sustained activation of HIF‐1α/2α upregulates iron uptake genes like TFR1 and DMT1 while suppressing the expression of hepatic hepcidin, ultimately resulting in systemic and local tumor iron overload [5]. Mechanistic studies have demonstrated that iron deprivation inhibits HIF‐α protein levels and transcriptional activity in RCC cells, and iron deprivation‐induced apoptosis and cell cycle arrest in RCC cells depend on VHL loss [14]. Although the role of iron overload in RCC cells is well established, its impact on other cells in the TME remains poorly understood.

RCC is one of the most common malignant tumors of the urinary system [15]. Among newly diagnosed kidney cancer patients, 20% present with metastatic renal cell carcinoma (mRCC), which responds poorly to surgery, radiotherapy, and chemotherapy, resulting in a poor prognosis and a 5‐year survival rate of less than 10% [16]. Currently, PD‐1 inhibitors are widely used in first‐line treatment for advanced RCC, yet a subset of patients remains poorly responsive [17, 18, 19, 20]. CD8+ T cells are the core effector cells of anti‐tumor immunity, and their cytotoxic function is the key mechanism of most effective cancer immunotherapies [21]. PD‐1 inhibitors temporarily restore the effector function of exhausted CD8+ T cells by blocking inhibitory signals (e.g., the PD‐1/PD‐L1 pathway), thereby enhancing anti‐tumor immune responses [22]. Recent studies have shown that CD8+ T cells in different states play distinct roles in renal cell carcinoma. In clear cell renal cell carcinoma, intratumoral infiltration of CXCL13+CD8+ T cells directly drives poor clinical outcomes and promotes the formation of immune escape structures [23]. The tissue localization characteristics of CD8+ T cells in the TME of RCC determine the exhaustion state of their clonotypes, thereby affecting tumor immune responses [24]. Systemic metabolic alterations (e.g., dietary interventions or disease‐induced metabolic dysregulation) can significantly modulate CD8+ T cell function, consequently impacting their mediated immune responses [25]. However, the effects of iron overload in CD8+T cells on tumor immunity remain poorly understood.

In this study, we revealed that iron overload promotes cancer progression by downregulating ZDHHC12, which is predominantly expressed in proliferating CD8+ T cells. Further experiments confirmed that under high‐iron conditions, ZDHHC12 downregulation helps maintain FDX1 stability, ultimately activating cuproptosis in CD8+ T cells and impairing their tumor‐killing function. Based on these findings, we developed lipid nanoparticles (Zdhhc12‐LNPs), with characterization showing an average size of 64.8 nm, zeta potential of −2.6 mV, high dispersity (PDI = 0.03), and 97.7% encapsulation efficiency. Cryo‐EM confirmed spherical morphology, while mass spectrometry verified mRNA purity and capping efficiency. Functionally, ZDHHC12‐LNPs restored FDX1 palmitoylation, rescued CD8+ T cell function, and synergized with PD‐1 inhibitors in preclinical renal cell carcinoma models without inducing hepatotoxicity or nephrotoxicity. In conclusion, we have developed a promising immunotherapeutic sensitizing material for RCC patients with iron overload.

## 2 ZDHHC12 Mediates Iron Overload‐Promoted RCC Progression

**FIGURE 1:**
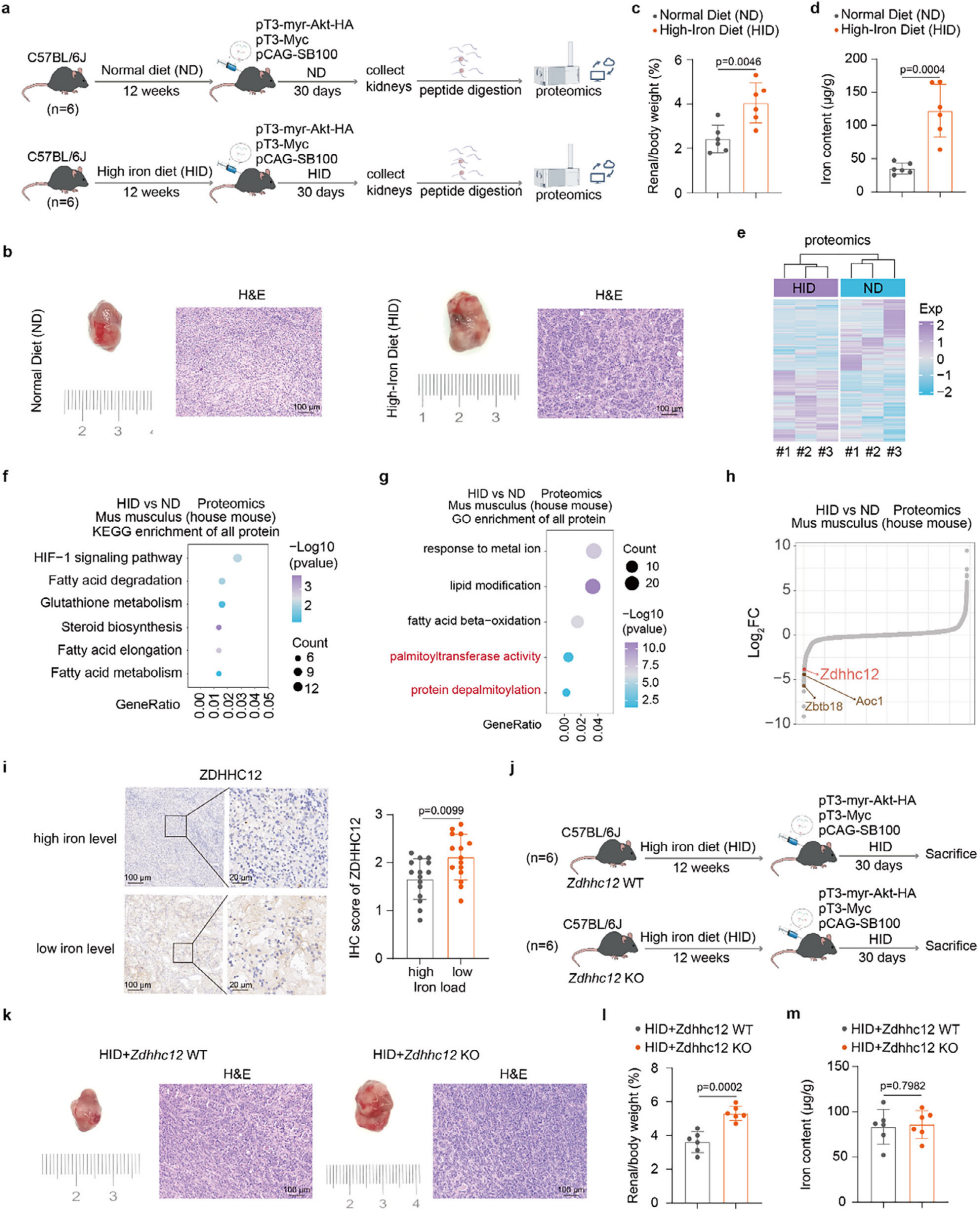
ZDHHC12 mediates iron overload‐promoted RCC progression. (a–d), Schematic diagram of mouse renal cancer models under different dietary feeding conditions (a). Specifically, after feeding mice with a high‐iron diet or a normal diet for 12 weeks, kidney plasmid injection was performed to establish the model. The original feeding regimen was continued for an additional 30 days, after which the kidneys were collected, and peptide segments from tumor tissues were extracted for proteomic analysis. Representative images of the kidney and H&E‐stained tumor tissue (b). The kidney‐to‐body weight ratio in renal cancer mice under different dietary feeding conditions reflects tumor burden (c). Samples were extracted from tumor tissues, and non‑heme iron content was analyzed in renal cancer mice fed different diets using a commercial ELISA kit (d). (e–h), Three renal cancer samples from the high‑iron diet group and three from the normal diet group were subjected to proteomic analysis (e), followed by KEGG enrichment analysis (f) and GO enrichment analysis (g) of the differentially expressed genes between the two groups. The fold‐change plot of differentially expressed genes in the high‐iron diet group compared to the normal diet group is shown, where each dot represents a gene and the vertical coordinate indicates its fold change (h). (i,j), Immunohistochemical staining was performed on renal cancer tissues from 15 patients with high iron load and 15 patients with normal iron load (i), and the expression of ZDHHC12 was statistically analyzed (j). (k–n), Zdhhc12 wild‐type or knockout genetically engineered mice were fed with a high‐iron diet, followed by kidney plasmid injection to establish a renal cancer model (k). Representative photographs of renal tumors and H&E‐stained tumor tissue sections from both groups are shown (l). The kidney‐to‐body weight ratio of tumor‐bearing mice in both groups reflects tumor burden (m). The non‐heme iron content in renal cancer tissues, reflecting tumor iron load, is also presented (n).

The impact of iron overload on RCC progression has long been a subject of interest, yet the underlying mechanisms remain poorly understood. In this study, we first observed that mice fed a long‐term high‐iron diet exhibited more pronounced RCC progression and higher serum iron levels compared to the normal diet group (Figure 1a–d). Subsequently, we conducted proteomic analysis on three randomly selected RCC samples from each group (normal diet and high‐iron diet) (Figure 1e and Figure S1a). Enrichment analysis revealed significant alterations in fatty acid metabolism and iron response‐related pathways (Figure 1f,g). Protein function is closely associated with post‐translational modifications, among which palmitoylation, a process dependent on palmitic acid, a key component of fatty acids, plays a crucial role [26]. Our enrichment analysis showed significant involvement of protein palmitoylation‐related processes (Figure 1g). Since protein palmitoylation relies on palmitoyltransferases, we examined these enzymes and found that only ZDHHC12 exhibited altered expression in high‐iron‐associated RCC tissues (Figure 1h). In patient samples, ZDHHC12 was significantly downregulated in tumor tissues from the high serum iron group compared to the low serum iron group (Figure 1i). To investigate the role of ZDHHC12 in RCC, we first modulated its expression in RCC cells to assess its effect on proliferation. The results showed that neither knockdown nor overexpression of ZDHHC12 affected RCC cell proliferation (Figure S1b,c). However, in *Zdhhc12* knockout mice subjected to a high‐iron diet, RCC progression was significantly enhanced compared to wild‐type mice (Figure 1j–m). In summary, our findings demonstrate that ZDHHC12 is a key driver in iron overload‐induced RCC progression.

## 3 Iron Overload Associated ZDHHC12 Primarily Participates in Maintaining the Activation of CD8+T Cells in Renal Cell Carcinoma

**FIGURE 2:**
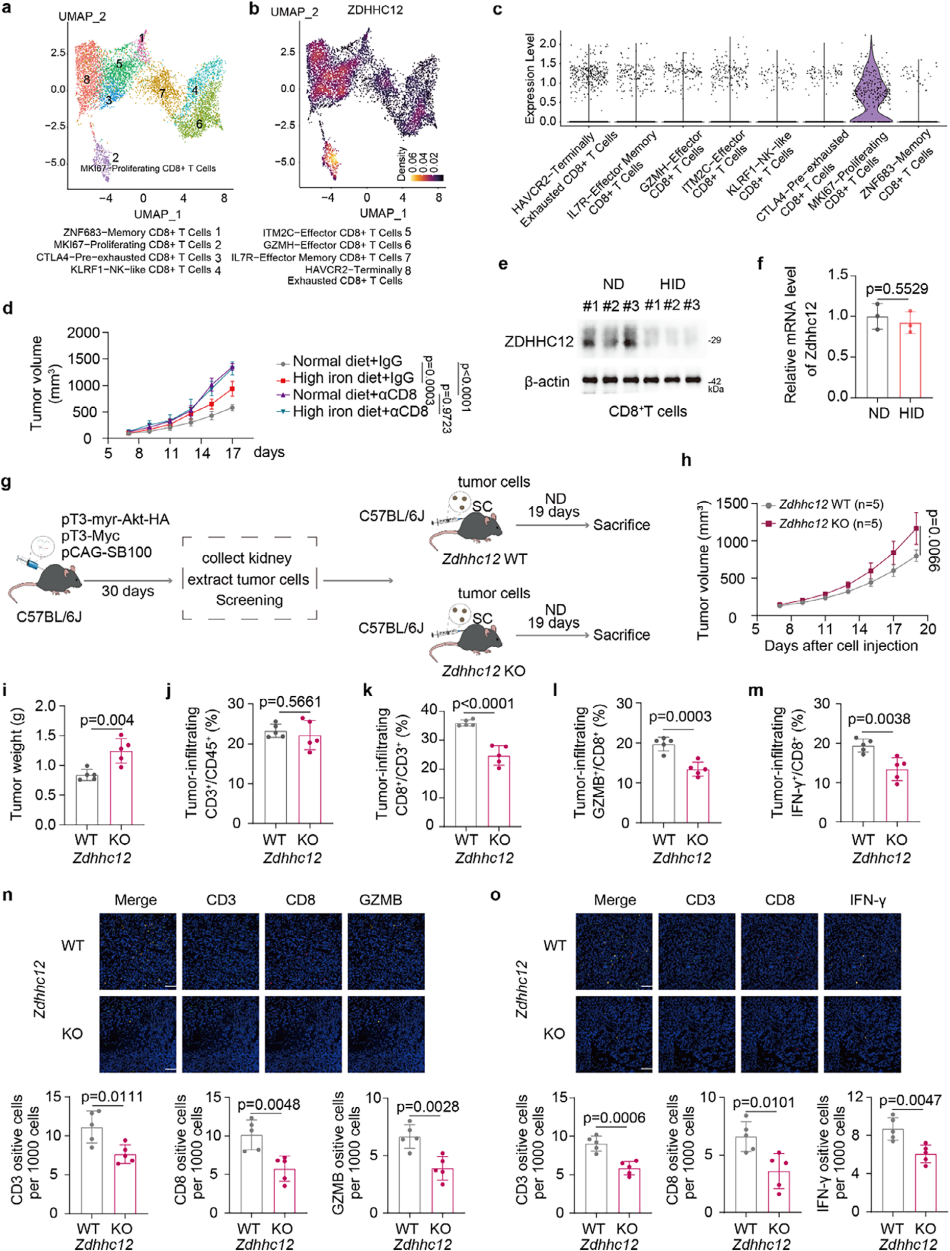
Iron overload‐associated ZDHHC12 primarily participates in maintaining the activation of CD8+T cells in renal cell carcinoma. (a) UMAP plot showing the subpopulation classification of CD8+ T cells (GSE121636). (b) UMAP visualization of ZDHHC12 expression across different CD8+ T cell subsets. (c) Violin plot depicting ZDHHC12 expression patterns in various CD8+ T cell subpopulations. (d) BALB/c mice fed either a high‐iron‐load diet or a normal diet were subcutaneously inoculated with Renca tumor cells on the dorsum, and starting from the day of inoculation, 200 µg of anti‐CD8 depletion antibody (αCD8) or an isotype control antibody was intraperitoneally administered every three days; tumor volume was measured from day 7 post‐inoculation. Data were presented as mean ± SD. e and f, Western blot (e) and RT‐qPCR (f) analyses were performed on CD8+ T cells sorted from renal cancer tissues of both the normal diet and high‐iron diet groups. (g–i), The figure illustrates schematic diagrams of different treatment groups (g). Specifically, tumor cells were isolated and sorted from the tumor tissues of the renal cancer model. Subsequently, the collected tumor cells were subcutaneously injected into the dorsum of either Zdhhc12 wild‑type or knockout mice. Tumor size was measured regularly, and growth curves were plotted (h). On day 19, the mice were sacrificed, and tumors were excised and weighed (i). (j–m), Portions of tumor tissues were processed for cell extraction, staining, and flow cytometry analysis to evaluate CD8+ T cell function in Zdhhc12 wild‐type and KO mice. Specifically, the proportions of CD3+/CD45+ (j), CD8+/CD3+ (k), GZMB+/CD8+ (l), and IFN‐γ+/CD8+ (m) T cells within tumor masses were quantified. (n,o) Portions of tumor tissues were embedded, sectioned, stained, and subjected to immunofluorescence analysis to assess CD8+ T cell function in Zdhhc12 wild‐type and KO mice. Specifically, multiplex staining was performed for DAPI/CD3/CD8/GZMB, and the proportions of positive cells were quantified (n); similarly, multiplex staining for DAPI/CD3/CD8/IFN‐γ was conducted with subsequent quantification of positive cell ratios (o).

Manipulation of ZDHHC12 did not regulate the malignant progression of RCC cells, but knockout of *Zdhhc12* significantly inhibited the tumor‐promoting effect of iron overload, suggesting that ZDHHC12 likely functions in cells other than tumor cells. CD8+ T cells are the core effector cells of anti‐tumor immunity, and their cytotoxic function is the key mechanism of most effective cancer immunotherapies [21]. Importantly, our rescue experimental results demonstrated that iron overload promotes tumor growth, but under conditions of CD8+ T cell depletion, iron overload did not further enhance tumor growth, indicating that CD8+ T cells are indispensable for the immunosuppressive effect of iron overload (Figure 2d and Figure S2b). Therefore, we next examined the expression distribution of ZDHHC12 in CD8+T cells. We found that the expression level of ZDHHC12 in proliferating CD8+T cells was significantly higher than in other cells (Figure 2a–c and Figure S2a). The Ki67+ proliferating CD8+ T cell subset represents an intermediate stage in the differentiation of CD8+ T cells into distinct lineages, and has been demonstrated to constitute an activated and tumor‐specific subpopulation within CD8+ T cells [27, 28]. In CD8+ T cells isolated from renal cancer tissues of high‐iron diet (HID)‐fed mice, the protein level of Zdhhc12 was significantly lower than that in the normal diet (ND) group (Figure 2e), while its RNA level remained unchanged (Figure 2f). The in vitro experimental results demonstrated that free iron decreases the percentages of GZMB and IFN‐γ in CD8+ T cells by downregulating ZDHHC12 (Figure S2c,d). We subsequently established subcutaneous tumor models to verify the impact of *Zdhhc12* knockout on anti‐tumor immunity (Figure S2e–g and Figure 2g). *Zdhhc12* knockout significantly promoted the volume growth (Figure 2h) and weight increase (Figure 2i) of RCC. We established the gating strategy for immune cell‐associated marker staining and flow cytometry analysis in tumors (Figure S3). *Zdhhc12* knockout significantly impaired infiltration of CD8+T cells, and the percentages of the cytokines GZMB and IFN‐γ (Figure 2j–o). Therefore, our results indicate that iron overload‐associated ZDHHC12 primarily participates in maintaining the activation of CD8+ T cells in renal cell carcinoma.

## 4 Iron Overload Promotes the Ubiquitin‐Proteasome Pathway Degradation of ZDHHC12 by Enhancing Its Binding With TRIM28

**FIGURE 3:**
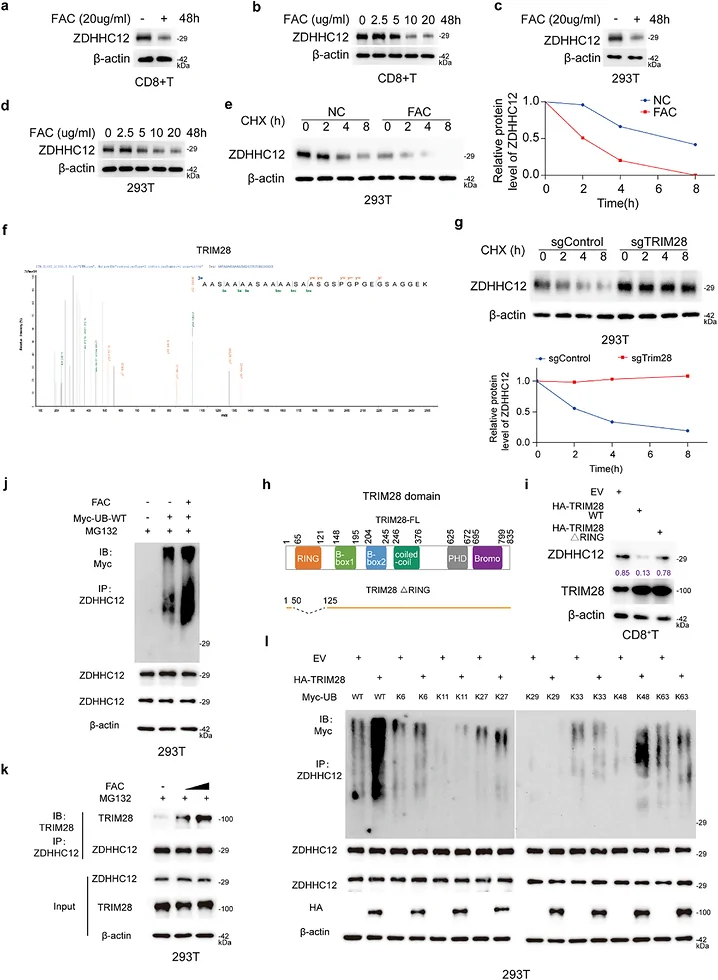
Iron overload promotes the ubiquitin‐proteasome pathway degradation of ZDHHC12 by enhancing its binding with TRIM28. (a) Cells were collected for Western blot analysis after treating sorted CD8+ T cells with FAC at the indicated concentrations for 48 h. (b) Cells were collected for Western blot analysis after treating sorted CD8+ T cells with FAC at the indicated concentration gradients for 48 h. (c) Cells were collected for Western blot analysis after treating 293T cells with FAC at the indicated concentrations for 48 h. (d) Cells were collected for Western blot analysis after treating 293T cells with FAC at the indicated concentration gradients for 48 h. (e) The degradation of ZDHHC12 in 293T cells was assessed by CHX chase assay after treatment with or without FAC (20 µg/mL) for 48 h. (f) HA‐ZDHHC12 was transfected into 293T cells, and co‐immunoprecipitation was performed using an anti‐HA‐ZDHHC12 antibody, followed by mass spectrometry to identify potential ZDHHC12‐interacting proteins. The peptide spectrum of TRIM28 is shown. (g) The degradation of ZDHHC12 in 293T cells was assessed by CHX chase assay with or without TRIM28 knockout. (h) Schematic diagrams depicting the protein domain architecture of TRIM28 and the TRIM28 △RING truncation mutant. (i) Sorted CD8+ T cells were stably transfected with the plasmids illustrated in the figure, followed by cell collection for Western blot analysis and quantitative assessment. (j) After transfection with the indicated plasmids, 293T cells were treated with FAC, followed by MG132 treatment, and then collected for Co‐IP and Western blot analysis. (k) After treatment with FAC (0, 20, 40 µg/mL), 293T cells were treated with MG132, collected, and subjected to Co‐IP and Western blot analysis. (l) After transfection with the indicated plasmids for 24 h, 293T cells were collected for Co‐IP and Western blot analysis.

Iron overload reduces ZDHHC12 expression levels, but the underlying mechanism remains unclear. We first confirmed in CD8+T cells that exogenous iron supplementation downregulates ZDHHC12 expression (Figure 3a). As exogenous iron concentration increased, ZDHHC12 protein levels decreased more significantly (Figure 3b). The same phenomenon was observed in 293T cells (Figure 3c,d). More pronounced ZDHHC12 degradation was observed in tool cells treated with exogenous iron (Figure 3e). To explore the mechanism of iron overload‐induced ZDHHC12 protein degradation, our protein mass spectrometry analysis revealed that TRIM28 may bind to ZDHHC12 (Figure 3f). TRIM28 possesses E3 ubiquitin ligase activity that can reduce protein stability [29]. We therefore hypothesized that iron overload promotes ZDHHC12 binding to TRIM28, ultimately leading to ZDHHC12 degradation. TRIM28 knockout significantly slowed ZDHHC12 degradation (Figure 3g). The RING finger domain of TRIM28 is a zinc finger motif that binds zinc ions via conserved histidine and cysteine residues, and this structure is essential for the E3 ubiquitin ligase activity of many TRIM proteins, enabling them to transfer ubiquitin molecules onto substrate proteins, thereby targeting them for proteasomal degradation [30]. We transfected CD8+ T cells with overexpression plasmids for wild‐type TRIM28 (HA‐TRIM28 WT) or a TRIM28 E3 ligase‐inactive mutant (HA‐TRIM28 △RING), followed by WB analysis to assess ZDHHC12 degradation (Figure 3h,i). The results showed that overexpression of TRIM28 alone was sufficient to degrade ZDHHC12, whereas overexpression of the E3 ligase‐inactive TRIM28 mutant (HA‐TRIM28 △RING) failed to degrade ZDHHC12 (Figure 3h,i). Exogenous iron supplementation increased both ZDHHC12‐TRIM28 binding and ZDHHC12‐ubiquitin binding (Figure 3j,k). TRIM28 promotes proteasome‐dependent ZDHHC12 degradation by mediating K48‐linked polyubiquitination (Figure 3l). In conclusion, we found that iron overload promotes the ubiquitin‐proteasome pathway degradation of ZDHHC12 by enhancing its binding with TRIM28.

## 5 ZDHHC12 Inhibits Cuproptosis in CD8+T Cells Through Interaction With FDX1

**FIGURE 4:**
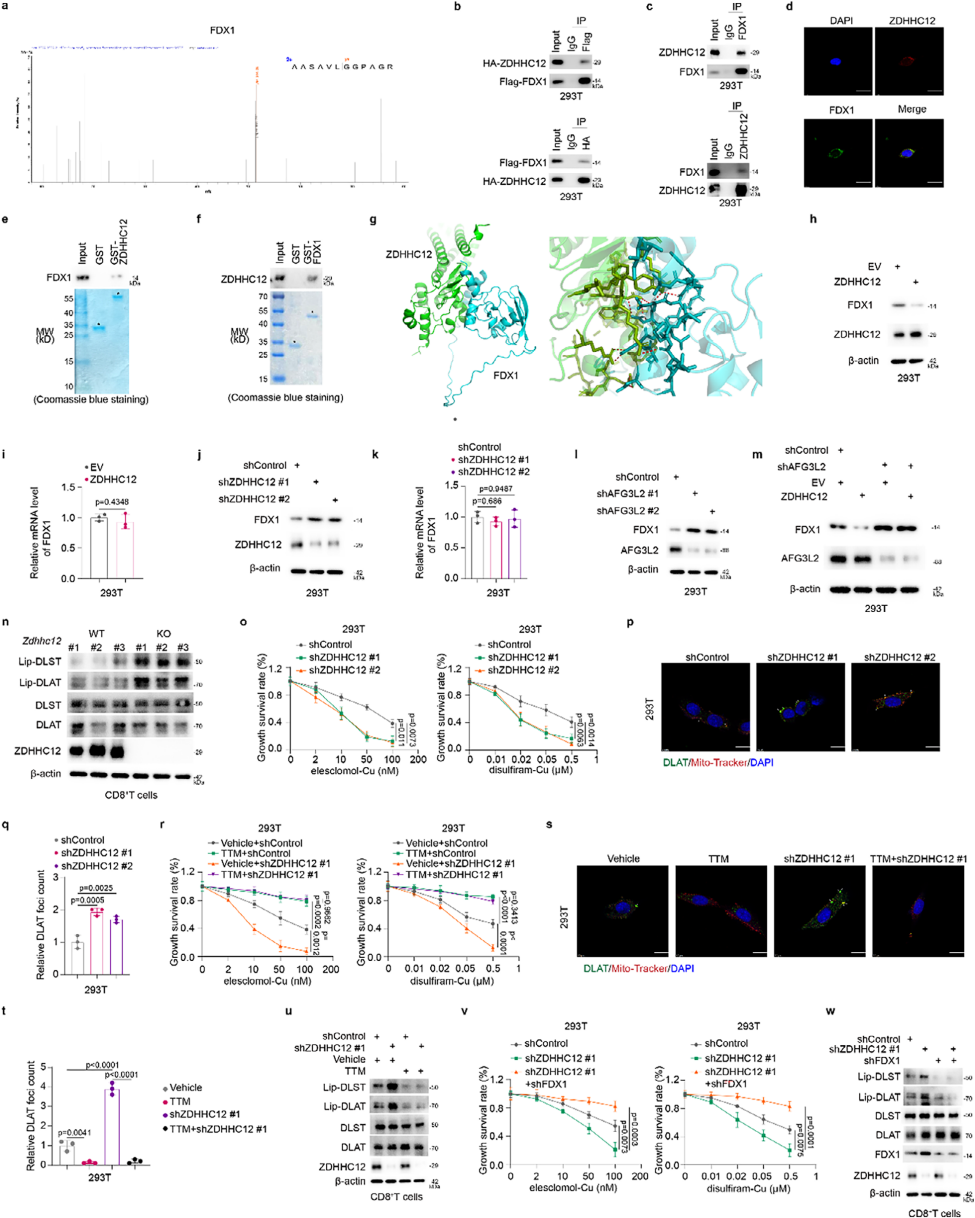
ZDHHC12 inhibits cuproptosis in CD8+T cells through interaction with FDX1. (a) We reviewed the mass spectrometry results and found that FDX1 may interact with ZDHHC12, and the peptide spectrum of FDX1 is shown in the figure. (b) HA‐ZDHHC12 and Flag‐FDX1 were transfected into 293T cells, followed by cell lysis and co‐immunoprecipitation using Flag or HA antibodies, and then analyzed by western blot. (c) 293T cells were lysed and subjected to co‐immunoprecipitation with ZDHHC12 or FDX1 antibodies, followed by western blot detection. (d) Immunofluorescence staining was performed in 293T cells using ZDHHC12 and FDX1 antibodies, and images were acquired and analyzed by confocal microscopy. (e) GST pull‐down assay was performed using the recombinant protein of ZDHHC12. (f) GST pull‐down assay was performed using the recombinant protein of FDX1. (g) Protein structure prediction and molecular docking were conducted for ZDHHC12 and FDX1. (h,i) Western blot (h) and RT‐qPCR (i) were performed in 293T cells after transfection with the indicated plasmids. (j,k) Western blot (j) and RT‐qPCR (k) were performed in 293T cells after transfection with the indicated plasmids. (l) Western blot analysis was conducted in 293T cells 72 h after transfection with the indicated plasmids. (m) Western blot analysis was performed in 293T cells after transfection with the indicated plasmids. (n) CD8+ T cells were isolated from renal cancer tissues of Zdhhc12 WT and Zdhhc12 KO mice and subjected to western blot analysis. (o) After successful transfection with the indicated plasmids in 293T cells, cells were collected and treated with different concentrations of elesclomol‐Cu or disulfiram‐Cu as shown in the figure for 48 h, followed by CCK‐8 assay to measure and quantify cell viability. (p,q) Following successful transfection with the indicated plasmids in 293T cells, cells were collected and immunostained with DLAT and Mito‐tracker, then imaged by confocal microscopy (p) to quantify (q) DLAT oligomerization. (r) After successful transfection with the indicated plasmids in 293T cells, cells were treated with different concentrations of elesclomol‐Cu or disulfiram‐Cu for 48 h in the presence or absence of tetrathiomolybdate (TTM) (1 µm) as indicated, followed by CCK‐8 assay to measure and quantify cell viability. (s,t) Following successful transfection with the indicated plasmids in 293T cells, cells were collected after treatment with or without tetrathiomolybdate (TTM) (1 µm), then immunostained with DLAT and Mito‐tracker and imaged by confocal microscopy (s) to quantify (t) DLAT oligomerization. (u) After successful transfection with the indicated plasmids in CD8+ T cells, cells were treated with or without tetrathiomolybdate (TTM) (1 µm), then collected for western blot analysis. (v) Following successful transfection with the indicated plasmids in 293T cells, cells were collected and treated with different concentrations of elesclomol‐Cu or disulfiram‐Cu as shown in the figure for 48 h, followed by CCK‐8 assay to measure and quantify cell viability. (w) After successful transfection with the indicated plasmids in sorted CD8+ T cells, cells were collected for western blot analysis.

ZDHHC12 is involved in iron overload‐induced renal cancer progression, but its mechanism of action remains unclear. We reviewed mass spectrometry results and found that in addition to TRIM28, ZDHHC12 may also bind to FDX1 (Figure 4a). FDX1 is the most cell viability‐ and cell death‐related protein among those potentially interacting with ZDHHC12. FDX1 is a key gene in the process of cuproptosis, which occurs in cancer as a newly discovered mode of cell death [31, 32]. Therefore, we speculated that ZDHHC12 might regulate cuproptosis in CD8+T cells through palmitoylation of FDX1. First, co‐immunoprecipitation, immunofluorescence co‐localization, GST‐pulldown, and molecular docking experiments all confirmed the binding between ZDHHC12 and FDX1 (Figure 4b–g). Subsequently, we found that knocking down ZDHHC12 upregulated FDX1 protein levels, while overexpressing it downregulated FDX1; however, ZDHHC12 manipulation did not affect FDX1 mRNA levels (Figure 4h–k). FDX1 is mainly located in the mitochondrial matrix, and studies have shown that the non‐classical protein degradation pathway mediated by the mitochondrial matrix‐related protease AFG3L2 leads to FDX1 degradation, while classical pathway protein degradation inhibitors such as MG132 and Baf‐A1 do not inhibit FDX1 degradation [33]. Therefore, we investigated whether ZDHHC12 affects the binding between AFG3L2 and FDX1, ultimately influencing its degradation. Knocking down AFG3L2 upregulated FDX1 protein levels (Figure 4l). Overexpression of ZDHHC12 reduced FDX1 protein levels, but overexpressing ZDHHC12 after AFG3L2 knockdown did not decrease FDX1 (Figure 4m). We then examined key indicators of cuproptosis. The lipoylation levels of DLAT and DLST in CD8+T cells from renal cancer in *Zdhhc12* KO mice were significantly higher than those in ZDHHC12 WT mice (Figure 4n). We found that knocking down ZDHHC12 enhanced the killing effect of cuproptosis inducers on cells and increased DLAT oligomerization (Figure 4o–q). We then found that the cuproptosis inhibitor (TTM) could reverse the increased cuproptosis caused by *Zdhhc12* knockdown (Figure 4r–u). Finally, knocking down FDX1 also reversed the increased cuproptosis caused by ZDHHC12 knockdown (Figure 4v,w). We also examined the mitochondrial ultrastructure of CD8+ T cells under different treatments using scanning electron microscopy, and the results showed that elesclomol‐Cu treatment induced mitochondrial shrinkage and cristae disappearance; knockdown of ZDHHC12 exacerbated mitochondrial shrinkage, cristae loss, and increased mitochondrial density; however, the mitochondrial damage caused by ZDHHC12 knockdown was reversed when FDX1 was concurrently knocked down (Figure S4a). In conclusion, our results demonstrate that ZDHHC12 interacts with FDX1 to promote its degradation through the mitochondrial matrix protease‐mediated non‐classical pathway, ultimately inhibiting cuproptosis.

## 6 ZDHHC12‐Mediated Palmitoylation of FDX1 at Cys152/155 Promotes Its Degradation

**FIGURE 5:**
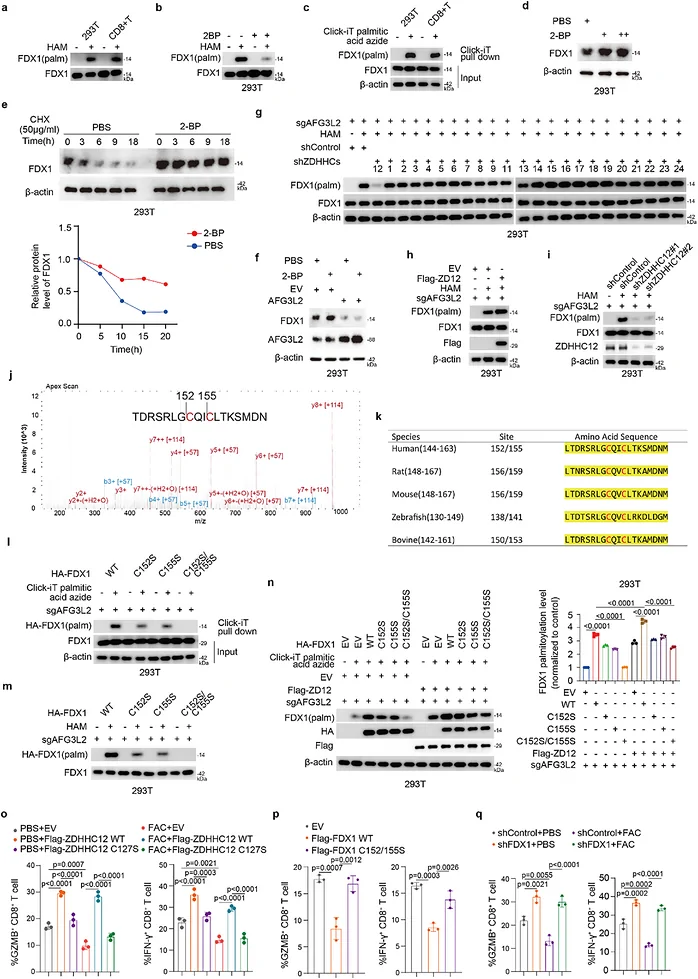
ZDHHC12‐mediated palmitoylation of FDX1 at Cys152/155 promotes its degradation. (a) In 293T and CD8+T cells, FDX1 was immunoprecipitated using an anti‐FDX1 antibody, followed by the acyl‐biotin exchange (ABE) assay performed either in the presence or absence of hydroxylamine (HAM) treatment, with subsequent streptavidin‐HRP pulldown of biotin‐conjugated proteins to specifically detect palmitoylated FDX1. (b) In 293T cells, FDX1 was immunoprecipitated using an anti‐ FDX1 antibody, followed by the acyl‐biotin exchange (ABE) assay performed with or without hydroxylamine (HAM) treatment, and subsequently enriched with streptavidin‐HRP to isolate biotin‐conjugated proteins, thereby enabling the detection of FDX1 palmitoylation levels in both 2‐BP (25 µm, 24 h)‐treated and untreated conditions. (c) 293T cells treated with or without palmitic acid azide were collected for Click‐IT reaction and streptavidin pulldown. (d) 293T cells were treated with different concentrations of 2‐BP (+, 20 µm; ++, 40 µm) for 24 h, then collected for western blot analysis. (e) 293T cells treated with or without 2‐BP (25 µm, 24 h) were exposed to CHX and collected at different time points for western blot analysis. (f) AFG3L2‐knockout 293T cells transfected with the indicated plasmids were subjected to ABE assay and western blot analysis with or without HAM treatment. (g) AFG3L2‐knockout 293T cells transfected with the indicated plasmids were subjected to ABE assay and western blot analysis with or without HAM treatment. (h) AFG3L2‐knockout 293T cells transfected with the indicated plasmids were subjected to ABE assay and western blot analysis with or without HAM treatment. (i) 293T cells transfected with the indicated plasmids were treated with or without 2‐BP (25 µm, 24 h) after 24 h, then collected for western blot analysis. (j) The peptide spectrum for FDX1 palmitoylation site identification. (k) Conservation sequences of FDX1 C152 and FDX1 C155 sites across different species. (l) AFG3L2‐knockout 293T cells transfected with the indicated plasmids were subjected to Click‐iT pull‐down assay and western blot analysis with or without palmitic acid azide treatment. m, AFG3L2‐knockout 293T cells transfected with the indicated plasmids were subjected to ABE assay and western blot analysis with or without HAM treatment. (n) AFG3L2‐knockout 293T cells transfected with the indicated plasmids were subjected to Click‐iT pull‐down assay and western blot analysis with or without palmitic acid azide treatment, followed by quantitative analysis of FDX1 palmitoylation levels. (o) CD8+ T cells were transfected with the indicated plasmids, then treated with PBS or FAC (20 µg/mL), collected, and stained with the indicated fluorescent dyes for flow cytometry analysis. (p) CD8+ T cells were transfected with the indicated plasmids, then collected and stained with the indicated fluorescent dyes for flow cytometry analysis. (q) CD8+ T cells were transfected with the indicated plasmids, then treated with PBS or FAC (20 µg/mL), collected, and stained with the indicated fluorescent dyes for flow cytometry analysis.

Although FDX1 is a potential downstream target of ZDHHC12, whether ZDHHC12 promotes FDX1 palmitoylation remains unknown. We found that FDX1 can be palmitoylated, and this palmitoylation is inhibited by 2‐BP (Figure 5a–d and Figure S5a). The palmitoylation inhibitor 2‐BP significantly enhances FDX1 stability and prolongs its degradation time (Figure 5e). 2‐BP increased FDX1 protein levels, but when AFG3L2 was overexpressed, 2‐BP failed to elevate FDX1 protein levels (Figure 5f). Knockdown of ZDHHC12 decreases FDX1 palmitoylation levels, whereas knockdown of other palmitoyltransferases does not (Figure 5g). Overexpression of ZDHHC12 increases FDX1 palmitoylation, while knockdown of ZDHHC12 reduces it (Figure 5h,i and Figure S5b,c). Through mass spectrometry analysis of palmitoylation sites, we identified C152 and C155 as FDX1 palmitoylation sites, both showing evolutionary conservation (Figure 5j,k). Compared to wild‐type FDX1, FDX1 C152S or C155S mutants exhibit reduced palmitoylation levels, while the FDX1 C152S/C155S double mutant shows undetectable palmitoylation when performing protein palmitoylation detection on HA‐pulled down FDX1 protein (Figure 5l,m and Figure S5d,e). Furthermore, overexpression of FDX1 C152S or C155S mutants results in lower palmitoylation levels compared to wild‐type FDX1, and overexpression of the C152S/C155S double mutant further reduces palmitoylation (Figure 5n). However, ZDHHC12 overexpression can enhance FDX1 palmitoylation under all these conditions (Figure 5n). The C127 residue in ZDHHC12's catalytic DHHC domain is essential for its enzymatic activity [34]—mutation of this residue abolishes function. Subsequent experiments demonstrated that exogenous iron decreases the percentages of GZMB and IFN‐γ in CD8+ T cells (Figure S5f,o). Overexpression of ZDHHC12 WT increases the percentages of GZMB and IFN‐γ in CD8+T cells, while ZDHHC12 C127S overexpression fails to do so (Figure 5o). Overexpression of FDX1 WT led to decreased percentages of GZMB and IFN‐γ in CD8+ T cells, whereas overexpression of FDX1 C152S/C155S did not (Figure S5g and Figure 5p). Exogenous iron reduces the percentages of GZMB and IFN‐γ in CD8+ T cells by upregulating FDX1 (Figure S5h and Figure 5q). In conclusion, ZDHHC12 palmitoylates FDX1 at C152/C155 sites to promote FDX1 degradation, and this palmitoyltransferase activity enhances cytotoxic factor production in CD8+T cells.

## 7 ZDHHC12‐LNP Rescues Iron Overload‐Induced PD‐1 Blockade Resistance in RCC

**FIGURE 6:**
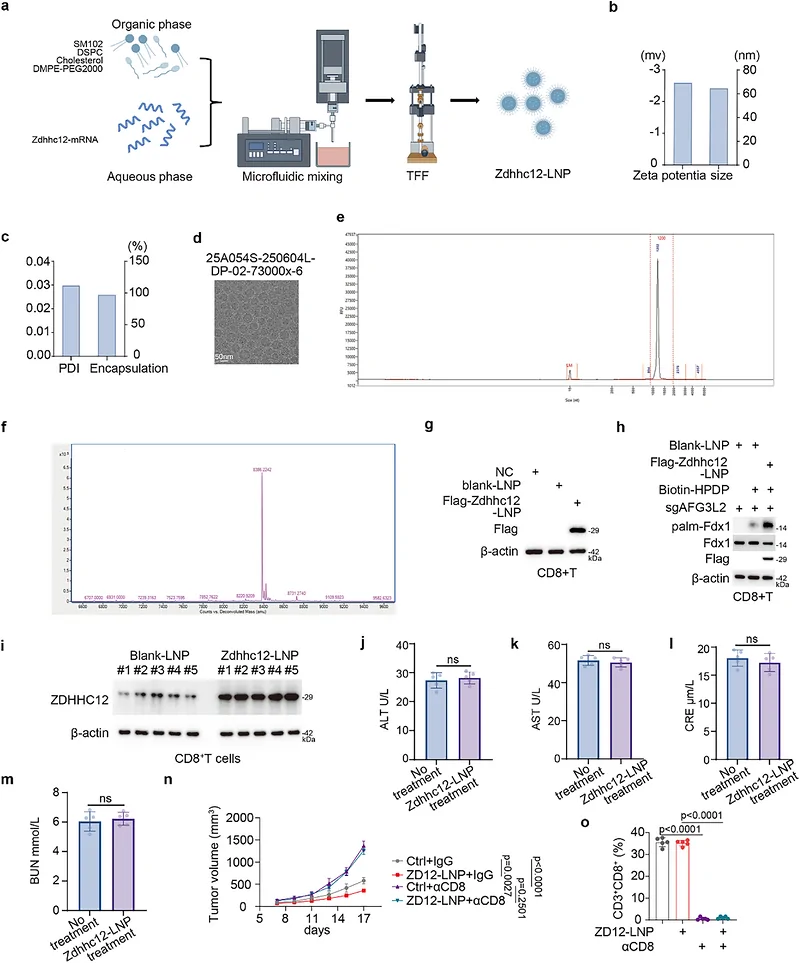
Development of lipid nanoparticles encapsulating Zdhhc12 mRNA (Zdhhc12‐LNP). (a) Schematic diagram of the LNP synthesis process; (b) The average zeta potential and the average particle size; (c) The average PDI and the average encapsulation efficiency; (d) Representative cryo‐EM image showing the morphology of synthesized LNPs. (e) Spectrum showing the purity of the sample. (f) Representative chromatogram showing mRNA capping efficiency. (g) Western blot analysis of Zdhhc12 expression in CD8+ T cells isolated from mice and treated with control, blank LNP, or Zdhhc12‐encapsulated LNP, following a 24 h pre‐stimulation with 2 µg/mL anti‐CD3/CD28 and subsequent 24 h incubation with LNPs. (h) ABE assay and Western blot analysis were performed on CD8+ T cells isolated from mice to assess the palmitoylation level of Fdx1, where cells were first cultured for 24 h post‐infection without anti‐CD3/CD28, restimulated with 2 µg/mL anti‐CD3/CD28 for 24 h, incubated with LNPs for 24 h, and then harvested for analysis. (i) Renca cells were subcutaneously injected into the backs of BALB/c mice, LNPs were administered via intratumoral injection, and when tumors reached an appropriate size, tumors were harvested for sorting of CD8+ T cells, followed by Western blot analysis to assess Zdhhc12 expression level in CD8+ T cells. (j–m) Renca cells were subcutaneously injected into the dorsal region of BALB/c mice. After tumor establishment, mice received intratumoral injection of LNPs or no injection, and serum levels of ALT (j), AST (k), CRE (l), and BUN (m) were measured at appropriate time points. Ns, not significant. (n,o) BALB/c mice were subcutaneously injected with Renca cells, intratumorally administered Zdhhc12‐LNP or control, and intraperitoneally injected with 200 µg anti‐CD8 (αCD8) or control; tumor growth was recorded starting from day 7 post‐tumor inoculation (n), and flow cytometry analysis of CD8+ T cell content in the peripheral blood of mice (n = 5) was performed at the end of the experiment (o), with data presented as mean ± SD.

We previously found that ZDHHC12 protein degradation under iron overload is a key factor in CD8+ T cell functional impairment. However, how to improve the efficacy of the most widely used cancer immunotherapy drug PD‐1 inhibitors, based on this discovery, still requires investigation. Given the current lack of ZDHHC12‐targeted activators, to address this challenge, we developed a lipid nanoparticle formulation (ZDHHC12‐LNP) using the established ionizable lipid SM‐102 along with DSPC, cholesterol, and DMG‐PEG2000 to encapsulate ZDHHC12 mRNA via microfluidic technology (Figure 6a). We first present a schematic diagram of the LNP synthesis process. Characterization results demonstrate that the prepared LNPs exhibit ideal physicochemical properties: an average zeta potential of −2.6 mV, an average particle size of 64.8 nm (Figure 6b), along with excellent dispersity (PDI = 0.03) and high encapsulation efficiency (97.7%) (Figure 6c). Cryo‐EM observation reveals that the LNPs display typical spherical structures (Figure 6d), while mass spectrometry analysis and chromatographic detection further confirm the high sample purity (Figure 6e) and mRNA capping efficiency (Figure 6f). Functional experiments show that Zdhhc12‐LNPs effectively transfect CD8^+^ T cells and significantly increase Flag‐Zdhhc12 expression levels (Figure 6g), while inducing marked changes in Fdx1 protein palmitoylation (Figure 6h). We treated subcutaneous tumor‐bearing mice with intratumoral injection of ZDHHC12‐LNP, subsequently sorted tumor‐infiltrating CD8^+^ T cells and tumor cells, and performed Western blot analysis; the results demonstrated that ZDHHC12‐LNP treatment significantly increased ZDHHC12 expression in both CD8^+^ T cells and tumor cells (Figure 6i and Figure S6a). Safety assessment revealed no significant differences in serum ALT (Figure 6j), AST (Figure 6k), CRE (Figure 6l), or BUN (Figure 6m) levels between Zdhhc12‐LNP‑treated tumor‐bearing mice and untreated controls, indicating that this nanoparticle system achieves efficient gene delivery without appreciably affecting liver or kidney function. To further investigate the in vivo biodistribution of the LNPs, we fluorescently labeled the LNPs with DiD and measured the fluorescence intensity in various tissues after intratumoral injection using a microplate reader; the results showed that the fluorescent signal was primarily retained in tumor tissue and lymph nodes, whereas only minimal signal was detected in the liver and spleen (Figure S6b). These findings indicate that intratumorally injected LNPs exhibit extremely limited off‐target distribution among organs in vivo, further supporting the favorable efficacy and safety profile of the LNP‐based intratumoral delivery strategy (Figure S6b). Our in vivo experimental results demonstrated that ZDHHC12‐LNP inhibited tumor growth; however, under conditions of CD8+ T cell depletion, this inhibitory effect was significantly attenuated, indicating that CD8+ T cells are essential for ZDHHC12‐LNP to exert its enhanced antitumor immune function (Figure 6n,o).

**FIGURE 7:**
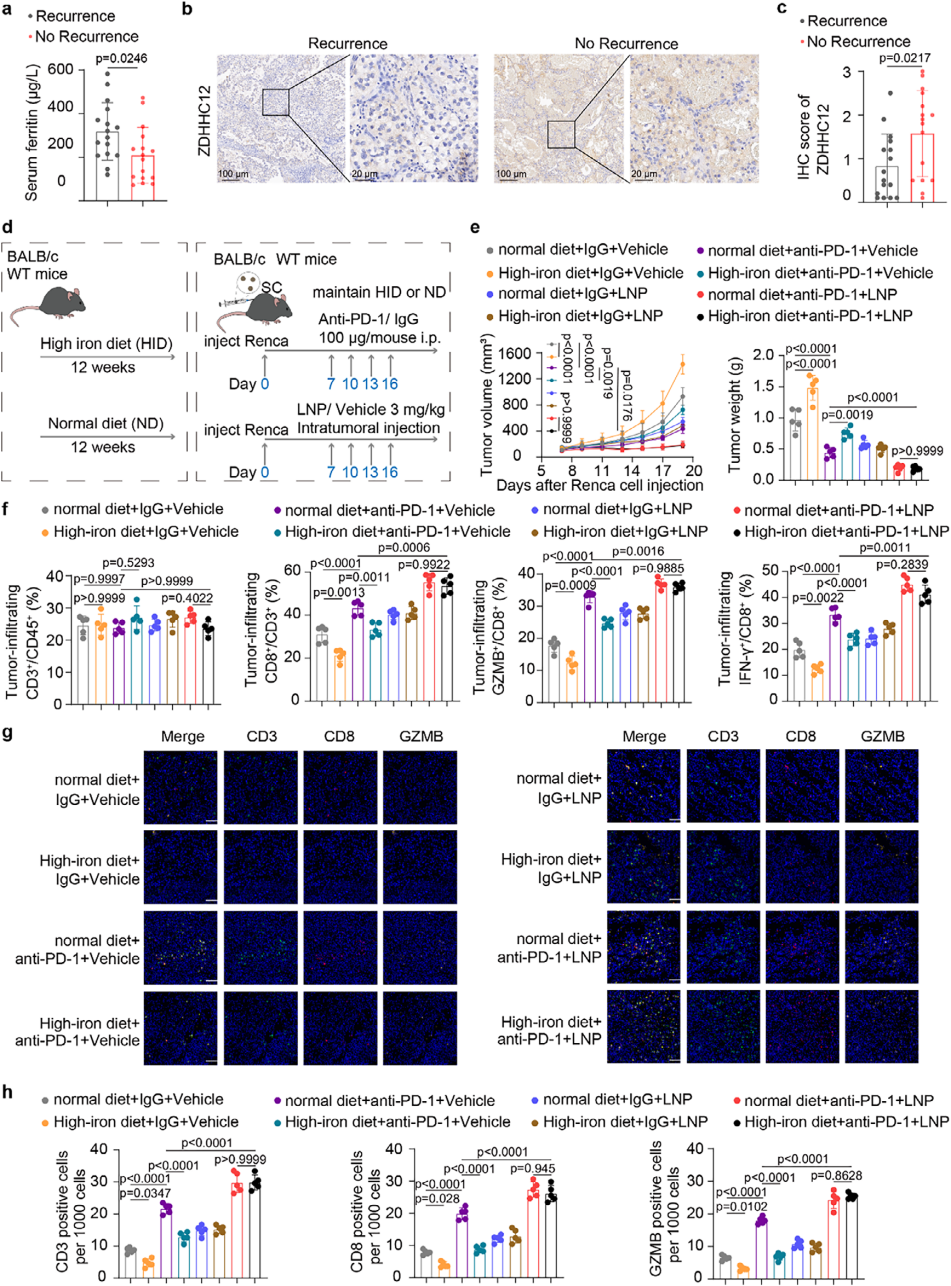
Zdhhc12‐LNP rescues iron overload‐induced PD‐1 blockade resistance in RCC. (a–c) We collected serum iron test results and pathological sections from patients who either experienced recurrence (n = 16) or remained recurrence‐free (n = 16) within 18 months after receiving adjuvant PD‐1 inhibitor therapy following renal cancer surgery. Each group consisted of 8 male and 8 female patients. We statistically analyzed and compared their serum iron levels (a), presented representative ZDHHC12 immunohistochemical staining images from both groups (b), and compared the immunohistochemical staining scores of ZDHHC12 between the two groups (c). (d–h) BALB/c mice fed with HID or ND were subcutaneously injected with equal amounts of Renca cells, then treated with anti‐PD‐1, IgG (control for anti‐PD‐1), Zdhhc12‐LNP, or Vehicle (control for Zdhhc12‐LNP) at the indicated doses and administration schedules on days 7, 10, 13, and 16 as shown in the figure (d). Specific treatment groups are detailed in the figure. Tumor volumes were measured regularly, and growth curves were plotted (e). Mice were euthanized at appropriate time points, and tumors were collected for weighing (e). Some tumor tissues were used for primary cell extraction, multiplex staining, and flow cytometry analysis (f). Some tumor tissues were subjected to multiplex immunofluorescence staining (g) for the indicated markers and statistical analysis of each marker's proportion (h).

**FIGURE 8:**
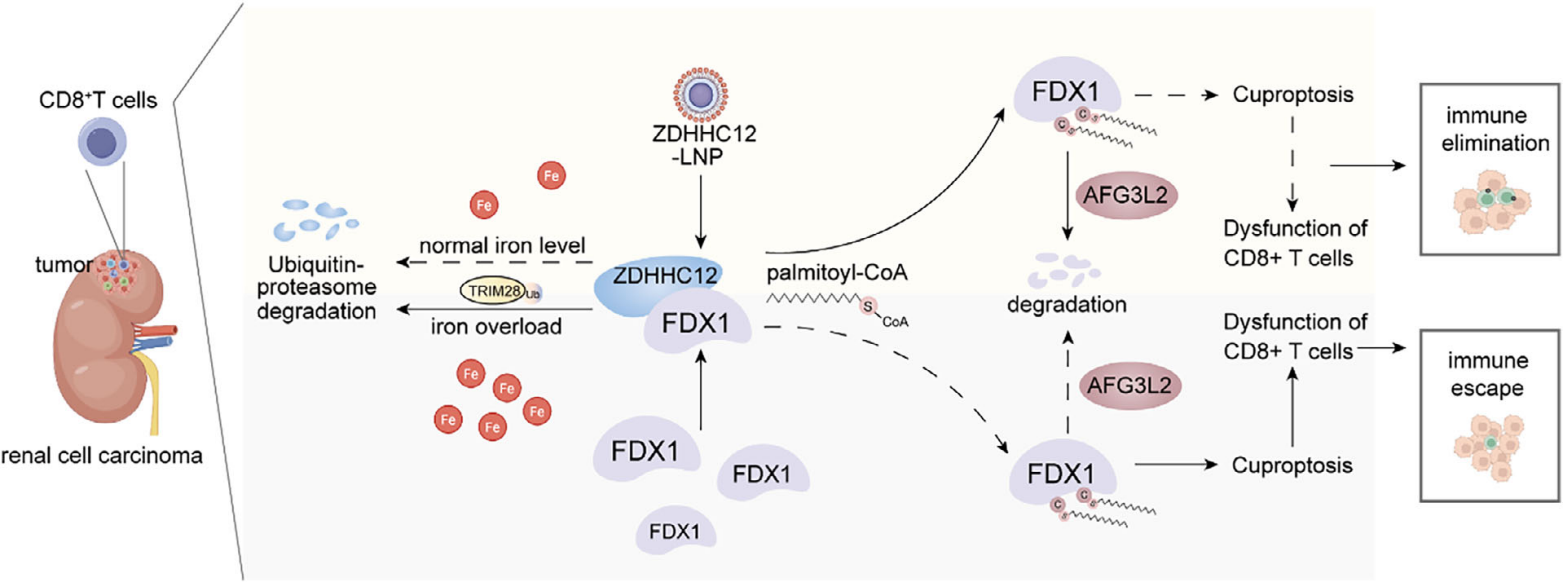
Under normal iron conditions, ZDHHC12‐mediated palmitoylation of FDX1 promotes FDX1 degradation by the mitochondrial matrix protease AFG3L2, ultimately suppressing cuproptosis in CD8+ T cells and maintaining their immune clearance function. Under iron overload conditions, increased binding between ZDHHC12 and TRIM28 leads to ZDHHC12 degradation, consequently inducing cuproptosis in CD8+ T cells and facilitating immune escape.

We performed immunohistochemical staining on tumor specimens from patients who received adjuvant PD‐1 inhibitor therapy after surgery. We found that patients with disease progression within 2 years post‐operation generally had iron overload and lower ZDHHC12 expression levels (Figure 7a–c). We then conducted combination therapy experiments with PD‐1 inhibitors and ZDHHC12‐LNP. The results demonstrated that iron overload diminished the anti‐tumor efficacy of PD‐1 inhibitors, but when ZDHHC12‐LNP was administered, the additional tumor‐promoting effects of iron overload were eliminated, and the therapeutic effect of PD‐1 blockade was enhanced (Figure 7d–h and Figure S7a,b). No significant differences were observed in body weight or liver/kidney function among the treatment groups after completion of the therapy (Figure S7c–g). In conclusion, our findings show that iron overload reduces PD‐1 inhibitor efficacy, and ZDHHC12‐LNP can improve this therapeutic outcome (Figure 8).

## 8 Outlook

PD‐1 inhibitors enhance anti‐tumor immune responses [22]. In this study, we discovered that iron overload suppresses antitumor immunity. Iron is an essential trace element involved in a wide range of fundamental biological processes, including oxygen transport, DNA synthesis, and cellular respiration [35]. Free iron catalyzes reactive oxygen species (ROS) production via the Fenton reaction, leading to lipid peroxidation, DNA double‐strand breaks, and gene mutations, which directly drive tumorigenesis [36]. Transferrin receptor 1 (TfR1), a critical mediator of iron uptake, is rapidly upregulated during early T‐cell activation [37]. A study has also shown that the distribution of T lymphocytes in various immune system compartments differs in rats under varying iron load conditions [38]. However, the regulatory role of iron overload in antitumor immunity remains poorly understood. Building on these findings, our study reveals a previously unrecognized mechanism in which iron overload induces copper‐dependent cell death (cuproptosis) in CD8+T cells, leading to impaired immune function and facilitating tumor immune evasion.

Proteomic analysis revealed that iron overload regulated pathways associated with lipid metabolism and protein palmitoylation. ZDHHC12 was the sole significantly altered palmitoyltransferase, leading us to hypothesize its involvement in iron overload‑driven renal cancer progression. ZDHHC12 is a palmitoyltransferase that mediates protein palmitoylation modifications, characterized by its essential DHHC domain responsible for palmitoyl group transfer [39]. Current evidence indicates its involvement in the progression and drug resistance of ovarian cancer and glioma [40, 41]. Furthermore, ZDHHC12‐mediated palmitoylation of NLRP3 can suppress inflammatory responses, which are typically closely associated with tumor progression [34, 42]. However, the distinct key downstream proteins of ZDHHC12 across various cancers contribute to the complexity of its functions in cancer; for example, ZDHHC12 stabilizes CLDN3 through palmitoylation to promote ovarian cancer progression [43]. Conversely, studies have shown that CLDN3 may inhibit the progression of renal cell carcinoma, lung squamous cell carcinoma, and colorectal cancer [44, 45, 46]. We found that ZDHHC12 plays a unique role in renal cell carcinoma, where it does not affect cancer cell proliferation, but iron overload promotes CD8^+^ T cell cuproptosis through ZDHHC12. Under conditions of iron overload, ZDHHC12 expression was significantly downregulated, leading to reduced palmitoylation of its substrate protein FDX1 and consequently promoting cuproptosis. To further elucidate this mechanism, we focused on FDX1, a critical regulator in the cuproptosis pathway, and found it to be highly responsive to iron overload in CD8+T cells. FDX1 is a mitochondrial iron‐sulfur protein that transfers electrons from NADPH to mitochondrial cytochrome P450 enzymes via ferredoxin reductase (FDXR), and is involved in the metabolism of steroids, cholesterol, and bile acids [47]. More importantly, FDX1 serves as a key upstream regulator of protein lipoylation in the cuproptosis pathway and reduces Cu^2^^+^ to the more toxic Cu^+^. Previous studies have shown that loss of FDX1 confers resistance to copper‐induced cytotoxicity [48] and that ferroptosis inducers promote cuproptosis by stabilizing mitochondrial FDX1 and enhancing protein lipoylation [33]. Our study demonstrates for the first time that iron overload directly upregulates FDX1 expression level, rendering CD8+T cells more susceptible to cuproptosis.

In this study, we encapsulated Zdhhc12 mRNA into SM‐102‐based lipid nanoparticles, which effectively elevated ZDHHC12 levels in CD8^+^ T cells both in vitro and in vivo, directly suppressing CD8^+^ T cell cuproptosis via palmitoylation of FDX1 and thereby promoting immune activation. Importantly, we found that this immunostimulatory effect was abolished upon CD8^+^ T cell depletion, underscoring the indispensable role of CD8^+^ T cells. ZDHHC12 did not promote malignant progression of renal cancer cells, and our LNPs exhibited favorable biosafety with minimal detectable off‑target distribution to other organs following local injection. Collectively, these results demonstrate that our Zdhhc12‑LNPs can safely activate antitumor immunity. However, several limitations remain. First, the impact of iron overload may extend beyond CD8^+^ T cells. In addition to CD8^+^ T cell‑mediated adaptive immunity, other innate immune cells in the tumor microenvironment can exert direct or indirect tumor‑killing functions and interact with CD8^+^ T cells to varying degrees [49, 50, 51, 52, 53]. The relationship between these cells and CD8^+^ T cells under iron‑overload conditions warrants further investigation; nevertheless, we have demonstrated that ZDHHC12 directly activates CD8^+^ T cells and that rescue experiments confirm the necessity of CD8^+^ T cells. Second, although we identified FDX1 as a key downstream target of ZDHHC12 in tumor‑infiltrating CD8^+^ T cells in renal cell carcinoma, other potential downstream effectors of ZDHHC12 remain to be explored. Third, while the molecular characteristics of ZDHHC12 allow non‑targeted Zdhhc12‑LNPs to activate antitumor immunity, we anticipate that developing CD8^+^ T cell‑targeted LNPs could further enhance antitumor immune efficacy. A recent study by Theresa L. Hunter et al. published in Science reported the specific targeting of CD8^+^ T cells by conjugating anti‑CD8 antibodies to L829‑tLNPs, providing a reference for our future work [54]. Fourth, although repeated local injections of LNPs showed good safety and no accumulation in other organs, we did not investigate the effects of sustained local administration or its impact on tumor recurrence. Qi et al. developed a flower‑shaped “RLNP” system that, when encapsulated in a photocrosslinked hyaluronic acid gel during surgery, enables sustained local drug release, significantly preventing postoperative tumor recurrence and extending animal survival [55], which offers a promising direction for our subsequent research.

In conclusion, we have delineated a novel immunosuppressive axis in which iron overload downregulates ZDHHC12, reduces FDX1 palmitoylation, and thereby activates cuproptosis in CD8+T cells, ultimately impairing antitumor immunity. We have identified ZDHHC12 as a promising therapeutic candidate target. Importantly, our lipid nanoparticle (LNP)‐encapsulated ZDHHC12‐targeting compound effectively restored CD8+T cell function under high‐iron conditions, offering a promising therapeutic strategy to counteract iron‐induced immune suppression and enhance the efficacy of cancer immunotherapy.

## 9 Methods

### 9.1 Chemicals and Reagents

Ferric ammonium citrate (FAC) (Cat# E0375, Selleck); Tetrathiomolybdate (TTM) (Cat# E1166, Selleck); 2‐BP (Cat# E0120, Selleck); biotin‐HPDP (#SML3797, Merck); streptavidin (Cat# 47503ES03, YEASEN); Click‐iT palmitic acid azide (Cat# C10265, Thermo Fisher Scientific). E. coli BL21 (Cat# C600003, Thermo Fisher Scientific). The sequences of all single guide RNAs (sgRNAs) and short hairpin RNAs (shRNAs) were listed in Table S1. The siRNAs were obtained from RiboBio (Guangzhou, China). The shRNAs and plasmids were purchased from GeneCopoeia (USA). Primary antibodies used were as follows: anti‐ZDHHC12 (Cat# ab237688, abcam), mouse monoclonal anti‐Myc tag (Cat# 60003‐2‐Ig, Proteintech), anti‐TRIM28 (Cat# 15202‐1‐AP, Proteintech), anti‐DLST (Cat #5556, cell signaling), rabbit polyclonal anti‐DLAT (Cat# 13426‐1‐AP, Proteintech), mouse monoclonal anti‐DLAT (Cat# 68303‐1‐Ig, Proteintech), anti‐FDX1 (Cat# 12592‐1‐AP, Proteintech), anti‐AFG3L2 (Cat# 14631‐1‐AP, Proteintech), rabbit polyclonal anti‐HA (Cat# 51064‐2‐AP, Proteintech), rabbit polyclonal anti‐Flag (Cat# 20543‐1‐AP, Proteintech), mouse monoclonal anti‐Flag (Cat# 66008‐4‐Ig, Proteintech), anti‐Beta Actin (Cat# 20536‐1‐AP, Proteintech). The reagents used for the immunofluorescence assay were Triton X‐100 (Cat# P0096, Beyotime), Mito Traker (Cat# C1035, Beyotime), CoraLite647‐conjugated F(ab')2 Fragment Goat Anti‐Rabbit IgG (H+L) (SA00014‐9, Proteintech) and CoraLite488‐conjugated Goat Anti‐Mouse IgG(H+L) (SA00013‐1, Proteintech).

### 9.2 Construction of Genetically Engineered Mice and Orthotopic Renal Cancer Models

All animal experimental procedures were approved by the Ethics Committee of the Second Xiangya Hospital of Central South University (Approval No.: 20250779). Mice were housed in an SPF‐level facility with a 12 h light/dark cycle under controlled temperature and humidity. The high‐iron diet group was fed standard AIN‐76A chow containing 50 mg iron/kg (Research Diets, Inc., New Brunswick, NJ). For tumor iron load in mice, non‑heme iron content was quantified using the Mouse FE (Ferritin) ELISA Kit (Cat# E‑EL‑M0491, Elabscience Biotechnology Co., Ltd.). To establish the orthotopic renal cancer model, we prepared a plasmid mixture containing 1 µg pT3‐AKT, 1 µg pT3‐Myc, and 0.2 µg pCAG‐SB100 in Ringer's solution. After anesthetizing mice via intraperitoneal injection of tribromoethanol, the lateral flank was disinfected with alcohol, and the skin/muscle layers were incised to expose the kidney. Without damaging renal tissue, the plasmid mixture was injected into the renal capsule using an insulin needle. The injection site was compressed with a cotton swab, the kidney was repositioned, and the wound was sutured in layers before returning the mice to their cages until sample collection. Zdhhc12 knockout mice (C57BL/6NCya‐Zdhhc12em1/Cya) were generated by Cyagen Biosciences using CRISPR/Cas9 technology. Cas9/gRNA complexes were microinjected into fertilized eggs of the C57BL/6NCya strain, targeting a 686‐bp region spanning exon 1 to exon 3 (including exon 2) for deletion.

### 9.3 Subcutaneous Tumor Growth and Treatment

Tumor cells were injected subcutaneously into the dorsal region of mice, and the longest (a) and shortest (b) diameters of the tumors were measured using a caliper to calculate tumor volume as ab^2^/2. For in vivo CD8+ T cell depletion experiments, 200 µg of anti‐CD8α antibody (MCE, HY‐P99129) was intraperitoneally administered every three days, with an equivalent amount of IgG isotype antibody as the control. For immune checkpoint blockade, 100 µg per mouse of anti‐PD‐1 antibody (Bioxcell, BE0146) or control IgG2α (Bioxcell, BE0085) was administered via tail vein injection. Treatment with Zdhhc12‐LNP and its control was administered via intratumoral injection at a dose of 3 mg/kg.

### 9.4 Mass Spectrometry and Proteomics Analysis

Mass spectrometry and proteomics analysis were performed with technical support from Shanghai Bioprofile Technology Co., Ltd. Mouse tissues were homogenized in lysis buffer (4% SDS, 100 mM DTT, 150 mM Tris‐HCl, pH 8.0), centrifuged (16,000 ×g, 15 min), and quantified by BCA. Proteins were digested via FASP: SDS removal (10 kDa cutoff), IAA alkylation (0.05 M, 20 min), tryptic digestion (1:50, 37°C, 20 h), and C18 desalting. Peptides were separated on a C18 column (75 µm×15 cm) with a 60‐min ACN gradient (2%–90%) at 300 nL/min, then analyzed by timsTOF Pro2 (PASEF mode, 100–1700 m/z, 42 eV). Data were processed using MSFragger/FragPipe against UniProt mouse DB (2021) with tryptic cleavage, fixed Cys alkylation, variable Met oxidation (1% FDR). Label‐free quantification used IonQuant.

### 9.5 Immunohistochemical Analysis

Tumor tissues from mice were obtained from our animal experiments, with all procedures approved by the Second Xiangya Hospital Ethics Committee (Approval No. 20250779). Human tissue samples were collected from the Chinese PLA General Hospital, with approval from the Hospital Ethics Committee (Approval No. S2021‐587‐03) and informed consent obtained from patients. Briefly, tissue blocks were paraffin‐embedded and sectioned, followed by deparaffinization, antigen retrieval, and blocking. The sections were then incubated with primary antibodies at 4°C overnight, followed by secondary antibody incubation and DAB visualization. Two experienced pathologists independently evaluated the IHC staining results. Staining intensity was graded as follows: Grade 1 (weak): faint staining visible at 40× magnification; Grade 2 (moderate): moderate staining at 40× magnification; Grade 3 (strong): intense staining at 40× magnification.

### 9.6 Cell Culture

HEK293T cells (ATCC, USA) were cultured in DMEM medium (Gibco) supplemented with 10% FBS (Gibco), with all media containing penicillin/streptomycin. Unless otherwise specified, “293T” mentioned in the figures and text refers to HEK293T. The cells were maintained in a humidified incubator at 37°C with 5% CO_2_. All cell lines were authenticated using short tandem repeat (STR) profiling and routinely tested to confirm the absence of mycoplasma contamination. Peripheral blood mononuclear cells were isolated from buffy coats of healthy donors by Ficoll gradient separation and were either used fresh or after cryopreservation in FBS supplemented with 10% dimethylsulfoxide (DMSO). The isolated PBMCs were resuspended and cultured in complete IMDM medium (Gibco) supplemented with 10% FBS.

### 9.7 Mouse Spleen Lymphocyte Isolation

Murine spleens were ground using a syringe plunger, filtered through a 40 µm strainer, and washed twice with PBS by centrifugation at 3000 rpm for 5 min. The cell pellet was resuspended in 1 mL of red blood cell lysis buffer, incubated at room temperature for 3 min, diluted with 9 mL of PBS, and centrifuged at 3000 rpm to obtain the cells.

### 9.8 Tumor Infiltrating Lymphocytes (TILs) Isolation

Tumor‐bearing mice were euthanized, and tumor tissues were completely excised. The tumors were then aseptically minced into approximately 1–3 mm^3^ fragments. The tissue fragments were transferred to a basal medium supplemented with 10% fetal bovine serum and 0.1 mg/mL collagenase I for dissociation. After digestion, the cell suspension was filtered through a 100 µm cell strainer. The filtrate was collected and washed twice with ice‐cold PBS. Cells were resuspended in 4 mL of 40% Percoll, and 3 mL of 80% Percoll was slowly added from the bottom of the tube. Centrifugation was performed at 3000 rpm for 20 min with an acceleration setting of 6 and a deceleration setting of 3. Following centrifugation, the cell layer at the interface between the two Percoll solutions represented the enriched tumor‐infiltrating lymphocytes. This layer was carefully aspirated, thoroughly washed with PBS, and high‐purity TILs were obtained.

### 9.9 In Vitro CD8+ T Cell Isolation and Stimulation

Murine CD8+ T cells were isolated from mouse‐derived cell suspensions using the MojoSort Mouse CD8+ T Cell Isolation Kit (BioLegend, #480008) according to the manufacturer's instructions and cultured in complete RPMI 1640 medium supplemented with 10% FBS and 50 µm β‐mercaptoethanol. Mouse T cells were stimulated using Dynabeads Mouse T‐Activator CD3/CD28 (Thermo Fisher, 11453D). Human CD8+ T cells were isolated from peripheral blood mononuclear cells (PBMCs) with the MojoSort Human CD8+ T Cell Isolation Kit (BioLegend, #480011) as per the manufacturer's protocol. Isolated human cells were cultured in X‑VIVO 15 Serum‑free Hematopoietic Cell Medium (Lonza) supplemented with 100 U/mL human IL‑2 (Peprotech). For human T cells, stimulation and expansion were performed using Human CD3/CD28 T Cell Activation Magnetic Beads (MCE, HY‑K0353).

### 9.10 Tumor Cell Sorting From Murine Tumor Tissues

Tumor cells were sorted from murine tumor tissues by rinsing the tissues with PBS on ice and mincing them. The tissue fragments were digested in tumor dissociation buffer (HY‐K6011, MedChemExpress) at 37°C. The resulting mixture was filtered through a 70 µm cell strainer to obtain a single‐cell suspension, which was treated with red blood cell lysis buffer (G2015‐500 mL, Servicebio) to remove erythrocytes and then resuspended in MACS buffer (BN24771, BIORIGIN) at a density of 1 × 10^8^ cells/mL. Negative magnetic bead sorting was subsequently performed. For every 1 × 10^7^ cells, 10 µL of anti‐mouse CD45 MicroBeads (Q0010, Selleck) were added, mixed thoroughly, and incubated at 4°C for 15 min with gentle flicking every 5 min to prevent aggregation. After incubation, 1 mL of MACS buffer was added, and the cells were centrifuged at 300 g for 3 min. The supernatant was discarded, and the pellet was resuspended in 1 mL of MACS buffer. An LD column was placed in a MACS separator magnet and equilibrated with 2 mL of MACS buffer. The cell suspension was then loaded onto the column, and the unlabeled flow‑through fraction was collected. The collected flow‑through was recounted, and for every 1 × 10^7^ cells, 10 µL of anti‐mouse CD31 MicroBeads (Miltenyi Biotec, 130‐097‐418) were added, mixed, and incubated at 4°C for 15 min with gentle flicking every 5 min. Following incubation, 1 mL of MACS buffer was added, and the cells were centrifuged at 300 g for 10 min. The supernatant was discarded, and the pellet was resuspended in 1 mL of MACS buffer. The suspension was then loaded onto a fresh LD column that had been equilibrated with 2 mL of MACS buffer, and the flow‑through was collected. The resulting CD31^−^CD45^−^ cell population was defined as tumor cells and used for subsequent experiments.

### 9.11 Lentivirus Production and Transfection

HEK293T cells were co‐transfected with the following plasmids using Lentifit transfection reagent (Hanbio, China): shRNA cloned into pLKO.1 vector, sgRNA cloned into lentiCRISPRv2 vector, or sequences cloned into pCDH vector, along with the helper plasmids (ΔR, VSV‐G, and Rev). Lentiviruses were harvested from the culture supernatant 48 h post‐transfection, filtered through 0.22 µm membranes to remove cell debris, and concentrated by ultracentrifugation at 25 000 ×g for 2 h at 4°C. The viral pellets were resuspended in serum‐free DMEM, aliquoted, and stored at −80°C for future use. For HEK293T cell transduction, HEK293T cells were seeded in appropriate culture dishes and infected with the packaged lentiviruses at 70%–80% confluency. After 6 h of incubation at 37°C, the medium was replaced with fresh complete medium, and cells were cultured for an additional 48 h before transfection efficiency analysis. Activated PBMC CD8+ T cells were plated at 1 × 10^6^ cells/well in 48‐well plates (400 µL X‐VIVO medium + IL‐2), infected with lentiviruses (MOI = 20), and centrifuged at 2500 rpm for 90 min to enhance infection efficiency. After 6 h, the medium was replenished to 1 mL/well, and cells were cultured for 72 h prior to transduction assessment. For cancer cells transduction, cells were seeded in appropriate culture dishes and infected with the packaged lentiviruses at 70%–80% confluency. After 6 h of incubation at 37°C, the medium was replaced with fresh complete medium. The sequence information used in transfection is provided in Table S1.

### 9.12 Flow Cytometry

For mouse samples, single‐cell suspensions from subcutaneous tumors were prepared through rapid and gentle dissection, followed by mechanical dissociation and filtration. Cells were first blocked with CD16/32 Pure Fc Block (BD Biosciences, 553141) to prevent nonspecific binding, and dead cells were excluded using Zombie Aqua Fixable Viability Kit (BioLegend, 423101). After washing, cells were resuspended in FACS buffer (PBS + 2% FBS) and surface‐stained for 30 min at 4°C with the following antibodies: APC‐Cy7‐CD45 (BD Biosciences, 561037), Pacific Blue‐CD11b (BioLegend, 101223), PE‐Cy7‐CD3 (BioLegend, 100319), PerCP‐Cy5.5‐CD8 (BD Biosciences, 561109), and Alexa Fluor 700‐CD4 (BD Biosciences, 561025). Following surface staining, cells were fixed and permeabilized using the Fixation/Permeabilization Solution Kit (BD Biosciences, 554714). After permeabilization, intracellular staining was performed with FITC‐GZMB (BioLegend, 515403) for granzyme B (GZMB) and APC‐IFN‐γ (BD Biosciences, 562018) for Interferon‐gamma (IFN‐γ). Stained cells were analyzed using a Cytek Northern Lights 3000 instrument, followed by data analysis with FlowJo software. For gating strategies, refer to Figure S2.

### 9.13 Western Blotting Analysis

RIPA lysis buffer (#G2002‐100ML, Servicebio, China) pre‐chilled to 4°C was supplemented with 1% protease inhibitor (#P1005, BioTeke, China) and 1% phosphatase inhibitor (#P1045, BioTeke, China) to maintain protein stability. After cell lysis on ice for 30 min, the lysate was centrifuged at 13 000 rpm for 15 min at 4°C, and the supernatant was collected. Protein concentration was determined using a BCA protein assay kit (#ab102536, Abcam, USA), followed by the addition of loading buffer and denaturation at 95°C for 10 min. The denatured samples were separated by SDS‐PAGE and transferred to a PVDF membrane. After blocking with skim milk for 1 h at room temperature, the membrane was incubated with primary antibodies overnight at 4°C, followed by secondary antibody incubation for 1 h at room temperature. Target protein signals were detected using ECL substrate (#34577, Thermo Fisher Scientific, USA).

### 9.14 Real‐Time Quantitative PCR (RT‐qPCR)

Total RNA was extracted from cells using TRIzol reagent (#AG21102, Accurate Biotechnology, China). RNA concentration and purity (A260/A280 ratio 1.8‐2.0) were measured using a NanoDrop2000 spectrophotometer (Thermo Fisher Scientific, USA). cDNA was then synthesized in a 20 µL reaction system using a reverse transcription kit (#AG11728, Accurate Biology, China). Subsequently, quantitative real‐time PCR was performed using the Evo M‐MLV One‐Step RT‐qPCR Kit (#AG11732, Accurate Biology, China) on a real‐time PCR system. All gene‐specific primers (sequences listed in Table S2) were synthesized by BGI (Beijing, China). Each sample was analyzed in triplicate, and the relative expression of target genes was calculated using the 2‐ΔΔCt method with β‐actin as the internal reference gene for normalization.

### 9.15 Immunofluorescence Staining

For cell samples, treated cells were seeded on confocal dishes, fixed with 4% paraformaldehyde for 15 min, permeabilized with 0.1% Triton X‐100 (in PBS) for 10 min, and blocked with 5% BSA. After overnight incubation with primary antibodies, samples were incubated with secondary antibodies for 1 h at room temperature. Nuclei were stained and mounted with DAPI‐containing mounting medium. For animal tissues, multiplex fluorescence staining was performed using a TSA multiplex kit (Servicebio, China). Paraffin sections were sequentially dewaxed in an eco‐friendly dewaxing solution, dehydrated in absolute ethanol, and rinsed with distilled water. Antigen retrieval was performed using an antigen retrieval instrument, microwave, or high‐pressure method, followed by natural cooling and washing with PBS on a shaker (3 × 5 min). After drying, sections were circled with a hydrophobic pen, rinsed with pure water or PBS, and incubated with 3% hydrogen peroxide for 25 min at room temperature in the dark to block endogenous peroxidase activity, followed by PBS washing (3 × 5 min). Sections were then blocked with 3% BSA or serum for 30 min at room temperature. After removing the blocking solution, primary antibodies were added and incubated overnight at 4°C in a humidified chamber. After PBS washing (3 × 5 min), HRP‐conjugated secondary antibodies were applied for 50 min at room temperature. Following another PBS wash, the TSA working solution (corresponding channel) was added and incubated for 10 min in the dark, followed by TBST washing (3 × 5 min). Antibody elution buffer was used for stripping. The staining and elution steps were repeated for multiplex labeling. Finally, DAPI staining, autofluorescence quenching, and mounting were performed before microscopic imaging.

### 9.16 Transmission Electron Microscopy (TEM)

The processed cells were collected and centrifuged, and the cell pellet was retained. One milliliter of pre‑cooled 2.5% glutaraldehyde fixative (#P1126, Solarbio, China) was slowly added to the pellet, and the cells were gently dispersed with a toothpick. After fixation in the dark at room temperature for 3–5 min, the mixture was transferred to 4°C for storage. Finally, ultrathin sections were examined by transmission electron microscopy (TECNA I20, Philips, Eindhoven, The Netherlands) to analyze mitochondrial morphological changes.

### 9.17 Acyl‐Biotin Exchange (ABE) Assay

Cell lysates were first incubated with 20 mM methanesulfonic acid (#23011, Thermo Fisher Scientific, USA) and 1 mM PMSF (#ST507, Beyotime, China) at 50°C for 30 min to completely block free thiol groups. Protein precipitation was then performed using cold acetone, followed by resuspension in 1 M hydroxylamine solution (pH 7.4, Sigma–Aldrich) to induce depalmitoylation. The treated proteins were subsequently labeled by incubation with 0.2 mM biotin‐HPDP (#SML3797, Merck, Germany) at room temperature for 1 h. Biotinylated proteins were finally isolated using streptavidin beads (#47503ES03, Yeasen, China) and analyzed by immunoblotting.

### 9.18 GST Pull‐Down Assay

GST‐tagged fusion proteins were expressed in E. coli BL21 (vector: pET‐GST) and extracted via lysis with muramidase followed by sonication. The lysate was incubated overnight at 4°C with glutathione‐agarose beads (#16100, Thermo Fisher Scientific, USA) to capture GST‐fusion proteins. After extensive washing, the bead‐bound GST‐fusion proteins were mixed with cell lysates containing the target protein and co‐incubated overnight at 4°C to allow interaction. The beads were then washed six times to remove nonspecifically bound proteins, and the complexes were eluted by boiling in SDS‐PAGE loading buffer for 10 min. The pulled‐down proteins were resolved by SDS‐PAGE and either stained with Coomassie Brilliant Blue (#G2059, Servicebio, China) for visualization or transferred to membranes for Western blotting to confirm specific interactions.

### 9.19 Co‐Immunoprecipitation (co‐IP)

Cells were lysed on ice for 30 min using Western/IP lysis buffer supplemented with protease and phosphatase inhibitors. The lysates were centrifuged at 13 400 × g for 10 min at 4°C to remove cellular debris. The supernatant was then incubated overnight at 4°C with primary antibody plus protein A/G agarose beads (#88802, Thermo Fisher Scientific, America) for antibody capture. After incubation, the beads were collected by centrifugation and washed six times with ice‐cold Western/IP lysis buffer to remove nonspecifically bound proteins. The immunocomplexes were eluted by boiling in 1× SDS‐PAGE loading buffer for 10 min at 95°C. The precipitated proteins were subsequently resolved by SDS‐PAGE and analyzed by western blotting.

### 9.20 Click‐iT Pull‐Down Assay

Cells were incubated with 100 µm Click‐iT palmitic acid azide (#C10265, Thermo Fisher Scientific, USA) for 6 h to metabolically label palmitoylated proteins. Following cell lysis and total protein extraction, the labeled proteins were conjugated to biotin‐alkyne using the Click‐iT Protein Reaction Buffer Kit (#C10276, Thermo Fisher Scientific, USA) according to the manufacturer's protocol. The biotinylated palmitoylated proteins were then precipitated with streptavidin beads (#47503ES03, Yeasen, China) at 4°C for 2 h. After extensive washing to remove nonspecifically bound proteins, the captured protein complexes were eluted by boiling in SDS‐PAGE sample buffer (without DTT) at 95°C for 10 min. The eluted proteins were subsequently resolved by SDS‐PAGE and analyzed by immunoblotting to identify specific palmitoylated proteins.

### 9.21 CCK‐8 Assay

Cell viability was assessed using the Cell Counting Kit‐8 (CCK‐8) assay (#C0037, Beyotime, China). Cells were seeded in 96‐well plates at a density of 8000 cells per well and allowed to adhere for 12 h. After treatment group assignment and appropriate interventions, 10 µL of CCK‐8 solution was added to each well and incubated at 37°C for 1 h. Absorbance was then measured at 450 nm using a microplate reader.

### 9.22 Proximity Ligation Assay (PLA)

Cells grown on coverslips (#FCP126, Beyotime, China) in 12‐well plates were fixed, permeabilized, and blocked with Duolink blocking solution. Primary antibodies were incubated overnight at 4°C, followed by PLA probe incubation (1 h). Ligation and amplification were performed using the Duolink in situ detection kit (Sigma‐Aldrich, USA), with washes between steps. Samples were mounted after final rinses and imaged.

### 9.23 Synthesis of Lipid Nanoparticles (Zdhhc12‐LNP)

The Zdhhc12 mRNA was pre‐dissolved in the aqueous phase. To prepare the lipid nanoparticle (LNP) formulation, an aqueous phase containing mRNA at a concentration of 0.35 mg/mL was prepared using 100 mM sodium acetate buffer (pH 4.0). Simultaneously, an organic phase was prepared by dissolving lipids in absolute ethanol at a total lipid concentration of 25 mM. The lipid components were as follows: SM102, Cholesterol, DSPC, DMG‐PEG 2000. The aqueous and organic phases were mixed at a volume ratio of 3:1 with a total flow rate of 12 mL/min using an Ignite microfluidic chip on a PNI Ignite instrument to encapsulate the plasmid into LNPs. The hydrodynamic diameter, zeta potential, and polydispersity index (PDI) of the LNPs were measured by Zetasizer Nano ZS. The morphology of LNPs was further examined by Cryo‐electron microscopy.

### 9.24 Bioinformatics Analyses

For downstream proteomic analysis, we performed differential expression analysis using the limma package. For enrichment analysis of differentially expressed genes, we utilized the clusterProfiler, org.Mm.eg.db, and org.Hs.eg.db packages with the Kyoto Encyclopedia of Genes and Genomes (KEGG) database. For single‐cell transcriptomic data analysis, we conducted comprehensive processing using the Seurat R package. After data preprocessing, we employed dimensionality reduction and clustering methods for cell classification. The Harmony package was applied to mitigate batch effects. The FindVariableFeatures function identified the top 2000 most variable genes. To ensure precise cell type annotation, we performed manual annotation.

### 9.25 Statistical Analysis

The experimental data were statistically analyzed using GraphPad Prism 7.0 software. For comparisons between different experimental groups, we used Student's *t*‐test, one‐way analysis of variance, or two‐way analysis of variance. All statistical results are expressed as mean ± standard deviation.

## Author Contributions

X.J., Y.L.H., C.L.Y. and W.Y.G. contributed equally. X.J., Y.L.H., C.L.Y. and W.Y.G. performed most of the experiments with help from R.J.Z., R.L.L., Z.X.D., X.L.L., S.Q.R., Q.Y.L. and Y.H.W. X.Z. provided support for clinical samples and experimental techniques. X.J., J.C., Y.L., X.M., and L.Y.G. designed the experiments. Y.L.H., R.J.Z., Q.Y.L., and Y.H.W. conducted bioinformatics analyses, including proteomics. C.L.Y., W.Y.G., and J.C. conducted the preparation and characterization of lipid nanoparticles. J.C., Y.L., X.M., and L.Y.G. guided and supervised the project. X.J., Y.L.H., C.L.Y., and W.Y.G. wrote the paper. All authors commented on the paper.

## Funding

This work was supported by the Chinese National Natural Science Foundation (Grant No. 82573476 (L.Y.G.); 82473229 (X.J.); 82373435 (Y.L.)), the Hunan Provincial Natural Science Foundation for Young Scholars (Class A) Continuation Project (2026JJ20010, X.J.), and The Science and Technology Innovation Program of Hunan Province (2025RC1020, Y.L.).

## Ethics Approval and Consent to Participate

The study was approved by the Chinese PLA General Hospital (Approval No. S2021‐587‐03) for patients’ samples and the Ethics Committee of the Second Xiangya Hospital of Central South University (Approval No. 20250779) for animals.

## Conflicts of Interest

J.C. is a co‐founder and shareholder of TargTex S.A. – Targeted therapeutics for Glioblastoma Multiforme. J.C. is a member of the Global Burden Disease (GBD) consortium of the Institute for Health Metrics and Evaluation (IHME), University of Washington (US), and is on the Scientific Advisory Board of Vector Bioscience Cambridge. The other authors have no conflicts of interest to declare.

## Supporting information




**Supporting File**: advs76022‐sup‐0001‐SuppMat.docx.

## Data Availability

The data that support the findings of this study are available on request from the corresponding author. The data are not publicly available due to privacy or ethical restrictions.
