# Supplementary material for: Materials‐Guided Gene‐Ionizable Lipid Nanoparticles to Reverse Iron‐Associated Immune Resistance in Renal Cancer
Source: Adv Sci (Weinh). 2026 Jun 9:e00078. Online ahead of print. doi: 10.1002/advs.202600078 (PMC13336895; doi:10.1002/advs.202600078)
Supplement: Supplementary file 1 — Supporting File: advs76022‐sup‐0001‐SuppMat.docx. [file ADVS-9999-e00078-s001.docx]

**Supplementary Information**

**Materials-guided gene-ionizable lipid nanoparticles to reverse iron-associated immune resistance in renal cancer**

Xin Jin^1,2*^, Yulong Hong^1,2*^, Chengliang Yin^3,*^, Wanyang Guo^1,2*^, Yaxuan Wang^4^, Ruijiang Zeng^1,2^, Ruilin Liu^1,2^, Zexian Ding^1,2^, Xinlin Liu^1,2^, Shangqing Ren^5^, Qiyang Liang^6^, Yaohui Wang^6^, Xu Zhang^6^, João Conde^7,#^, Yuan Li^1,#^, Xin Ma^5,#^, Liangyou Gu^6,#^

^1^ Department of Urology, The Second Xiangya Hospital, Central South University, Changsha, Hunan 410011, China.

^2^ Uro-Oncology Institute of Central South University, Changsha, Hunan 410011, China.

^3^ Department of Biomedical Engineering, Faculty of Engineering, University Malaya, Kuala Lumpur, 50603, Malaysia.

^4^ Department of Urology, The First Affiliated Hospital of Harbin Medical University, Harbin, 150001, China.

^5^ Robotic Minimally Invasive Surgery Center, Sichuan Provincial People's Hospital, School of Medicine, University of Electronic Science and Technology of China, Chengdu, 610072, China.

^6^ Department of Urology, Chinese PLA General Hospital, Beijing 100039, China.

^7^ Comprehensive Health Research Centre (CHRC), NOVA Medical School, Faculdade de

Ciências Médicas, NMS|FCM, Universidade NOVA de Lisboa, Lisboa, Portugal.

* These authors contributed equally to this work

^#^ Correspondence: [joao.conde@nms.unl.pt](mailto:joao.conde@nms.unl.pt) (J.C.), [yuanlixy@csu.edu.cn](mailto:yuanlixy@csu.edu.cn) (Y.L.), [urologist@foxmail.com](mailto:urologist@foxmail.com) (X.M.), [guliangyouyd1@126.com](mailto:guliangyouyd1@126.com) (LY.G.)

**
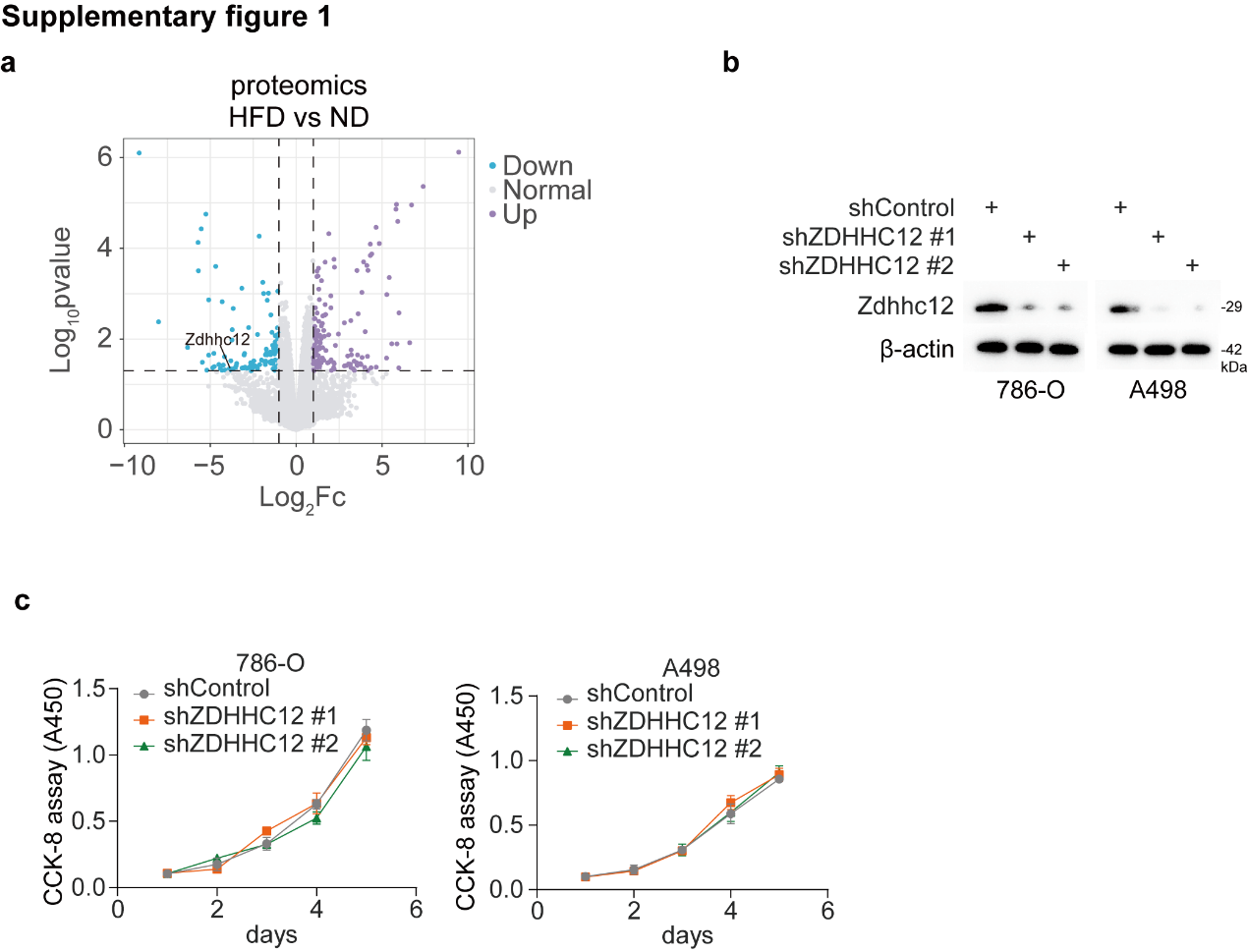
**

**Supplementary figure 1 (related to figure 1)**. **a**, Three renal cancer samples from the high‑iron diet group and three from the normal diet group were subjected to proteomic analysis. **b-c**, The indicated constructs were transfected into 786-O and A498 cells, and then the cells were collected for western blot (b) and CCK-8 assays (c).


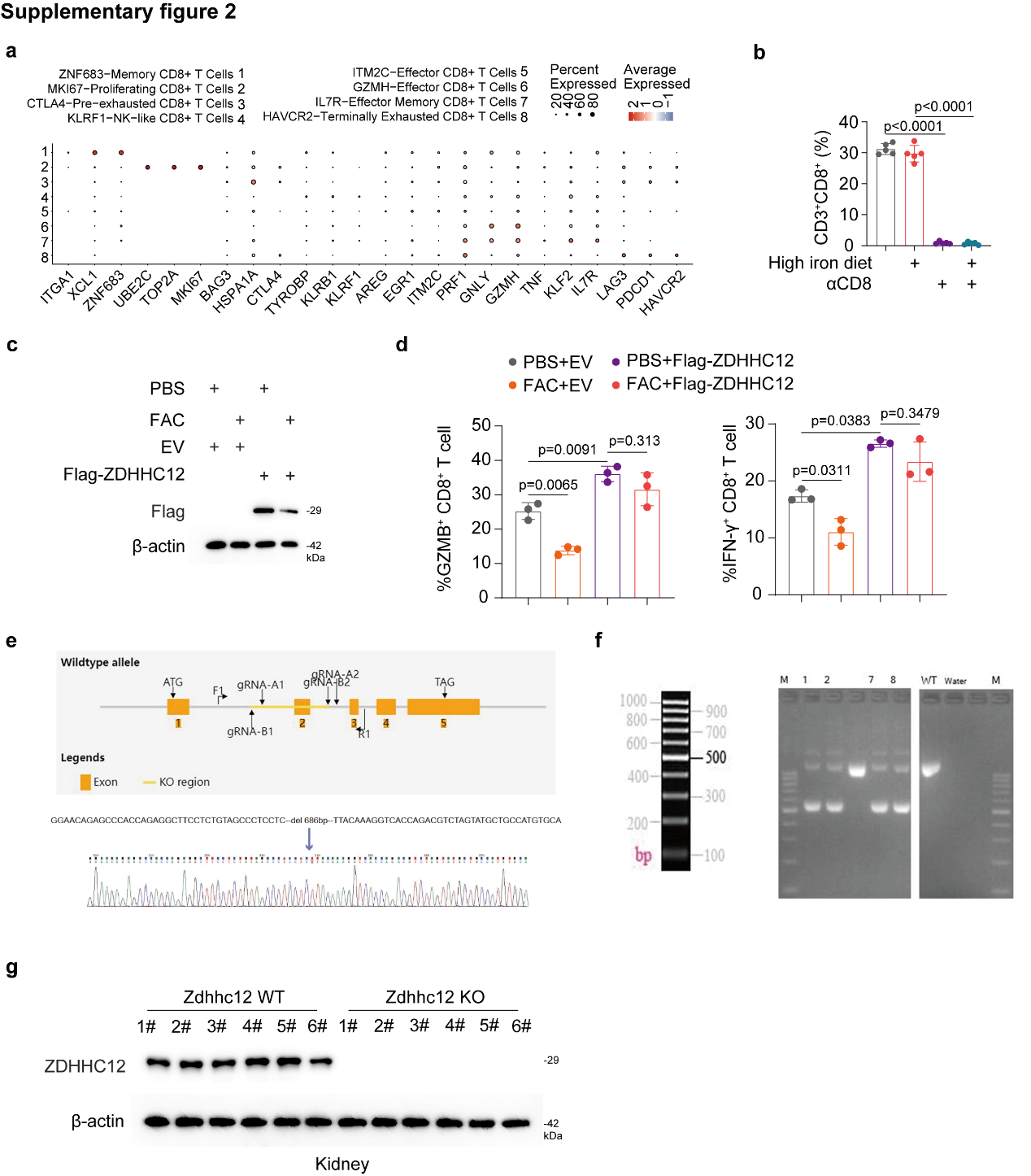


**Supplementary figure 2 (related to figure 2)**. **a**, The various CD8+ T cell subsets and their marker genes in the GSE121636 dataset. **b**, BALB/c mice fed either a high-iron-load diet or a normal diet were subcutaneously inoculated with Renca tumor cells on the dorsum, and starting from the day of inoculation, 200 μg of anti-CD8 depletion antibody (αCD8) or an isotype control antibody was intraperitoneally administered every three days; flow cytometry analysis of CD8+ T cell content in the peripheral blood of mice (n = 5) was performed at the end of the experiment, with data presented as mean ± SD. **c-d**, CD8+ T cells were transfected with the indicated plasmids, then treated with either FAC or PBS, and finally subjected to western blot analysis (c) and flow cytometry detection (d). **e**, A schematic diagram of the production of *Zdhhc12* KO mice and a demonstration of the knockout sequence. **f,** The tail end of the mouse was cut about 0.5 cm, and the tissue was lysed to extract DNA, which was amplified by PCR and then electrophoresed to identify the genetically engineered mice. Our genotyping results confirmed that mice #1, #2, #7, and #8 were positive for the targeted deletion (574-bp band), whereas the lanes between #2 and #7 represented mice negative for the deletion (wild-type or unsuccessful knockout, 1260-bp band), which were excluded from subsequent experiments. **g**, Western blot analysis of Zdhhc12 protein expression in kidneys of Zdhhc12 wild-type and Zdhhc12 knockout mice.


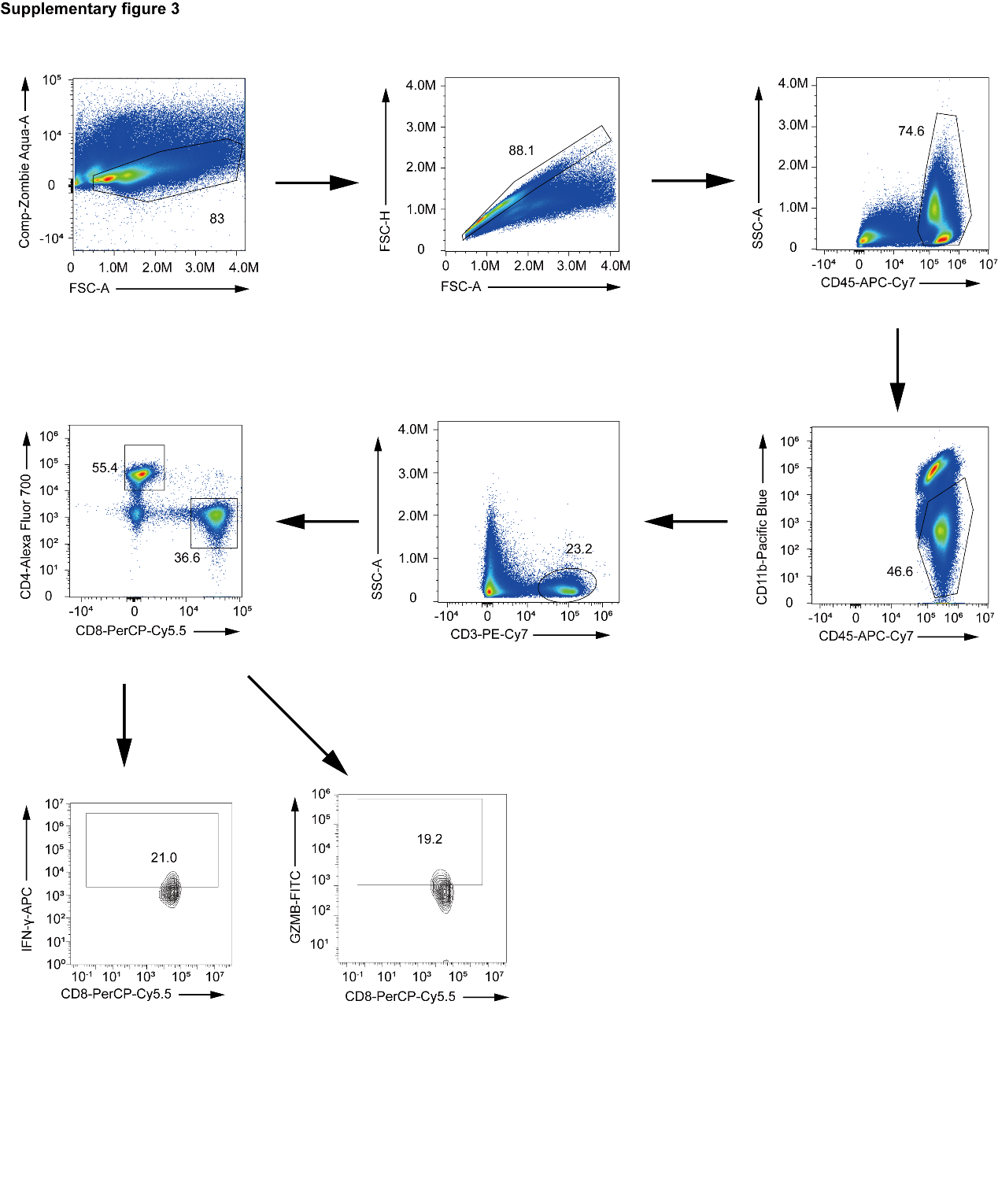


**Supplementary figure 3 (related to figure 2)**. Gating strategy to sort for CD3/ CD8/GZMB (IFN-γ) positive cells.


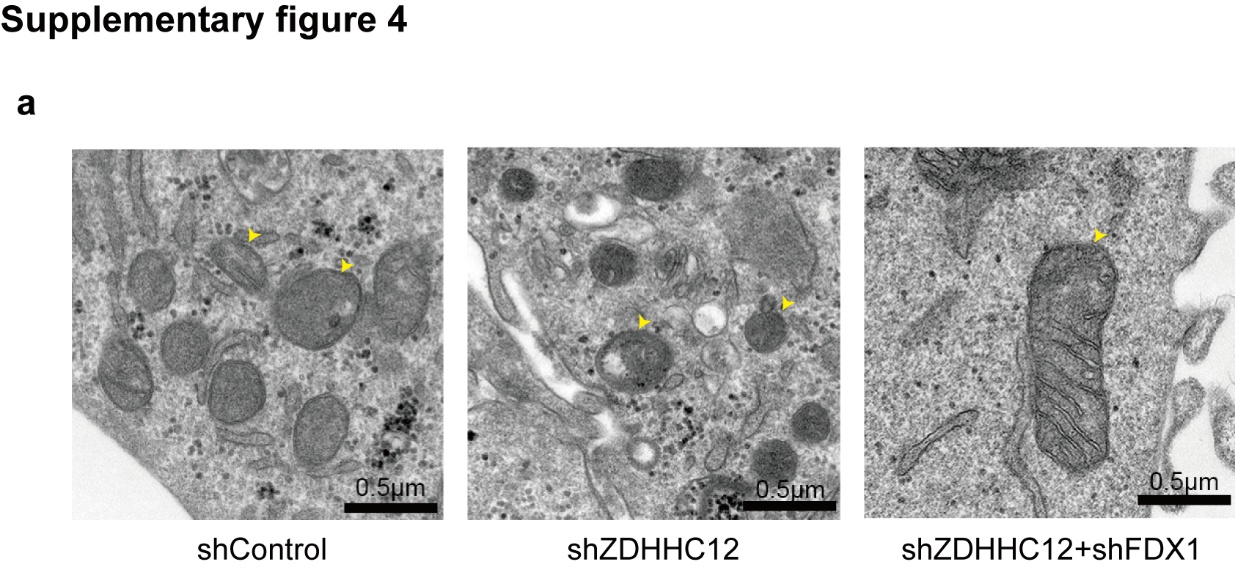


**Supplementary figure 4 (related to figure 4)**. **a**, Transmission electron microscopy (TEM) revealed the ultrastructural features of mitochondria in CD8+ T cells stably transfected with different shRNAs and treated with elesclomol-Cu (50 nM, 48 hours), with yellow arrows indicating mitochondria.


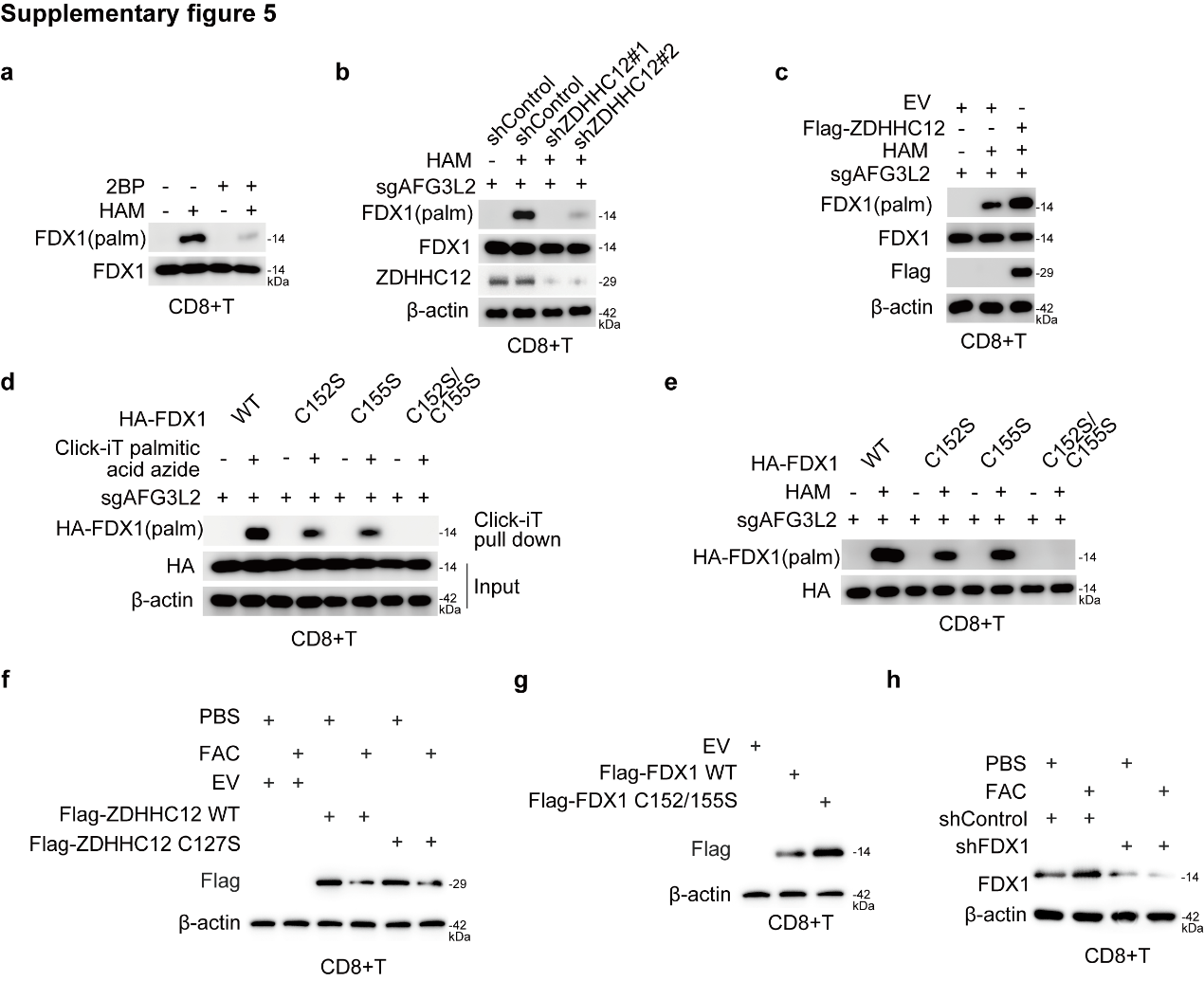


**Supplementary figure 5 (related to figure 5)**. **a**, In CD8+T cells, FDX1 was immunoprecipitated using an anti-FDX1 antibody, followed by the acyl-biotin exchange (ABE) assay performed with or without hydroxylamine (HAM) treatment, and subsequently enriched with streptavidin-HRP to isolate biotin-conjugated proteins, thereby enabling the detection of FDX1 palmitoylation levels in both 2-BP (25 μM, 24 hours)-treated and untreated conditions. **b**, AFG3L2-knockout CD8+ T cells transfected with the indicated plasmids were subjected to ABE assay and western blot analysis with or without HAM treatment. **c**, AFG3L2-knockout CD8+ T cells transfected with the indicated plasmids were subjected to ABE assay and western blot analysis with or without HAM treatment. **d**, AFG3L2-knockout CD8+ T cells transfected with the indicated plasmids were subjected to Click-iT pull-down assay and western blot analysis with or without palmitic acid azide treatment. **e**, AFG3L2-knockout CD8+ T cells transfected with the indicated plasmids were subjected to ABE assay and western blot analysis with or without HAM treatment. **f**, CD8+ T cells were transfected with the indicated plasmids, then treated with PBS or FAC (20 μg/mL), collected, and subjected to western blot analysis. **g**, CD8+ T cells were transfected with the indicated plasmids, then collected and subjected to western blot analysis. **h**, CD8+ T cells were transfected with the indicated plasmids, then treated with PBS or FAC (20 μg/mL), collected, and subjected to western blot analysis.


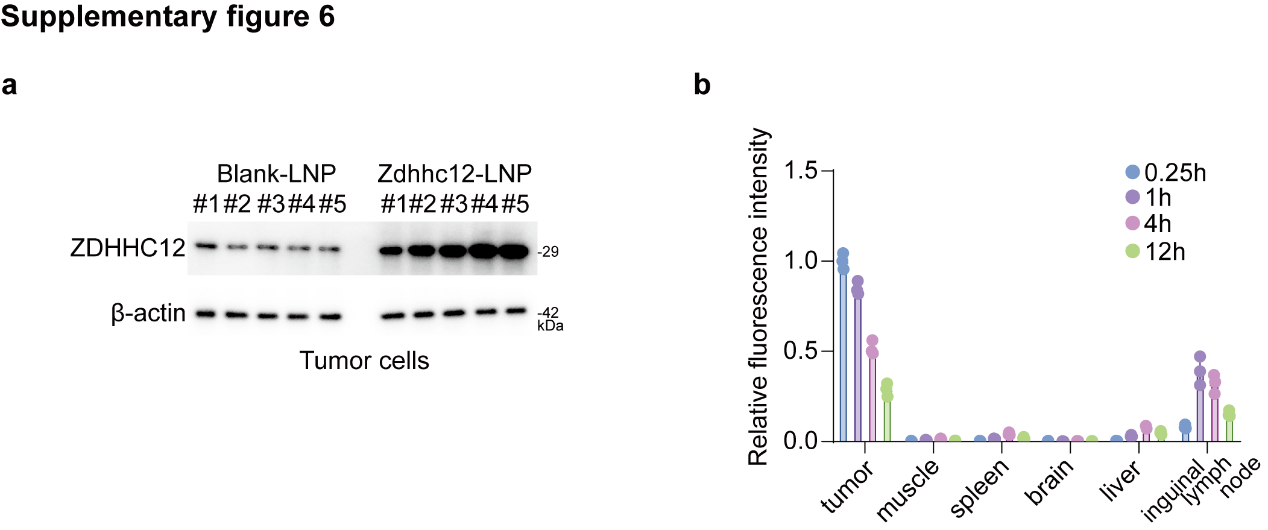


**Supplementary figure 6 (related to figure 6)**. **a.** Renca cells were subcutaneously injected into the backs of BALB/c mice, LNPs were administered via intratumoral injection, and when tumors reached an appropriate size, tumors were harvested for sorting of tumor cells, followed by Western blot analysis to assess Zdhhc12 expression level in tumor cells. **b**, BALB/c mice were subcutaneously injected with Renca cells, the LNPs were labeled with DiD, and after the appropriate time for tumor formation, Zdhhc12-LNP was administered intratumorally; tumors, lymph nodes, liver, spleen, skeletal muscle, and brain were collected at 0.25 h, 1 h, 4 h, and 12 h post-injection, and the fluorescence intensity in each tissue was measured using a microplate reader.


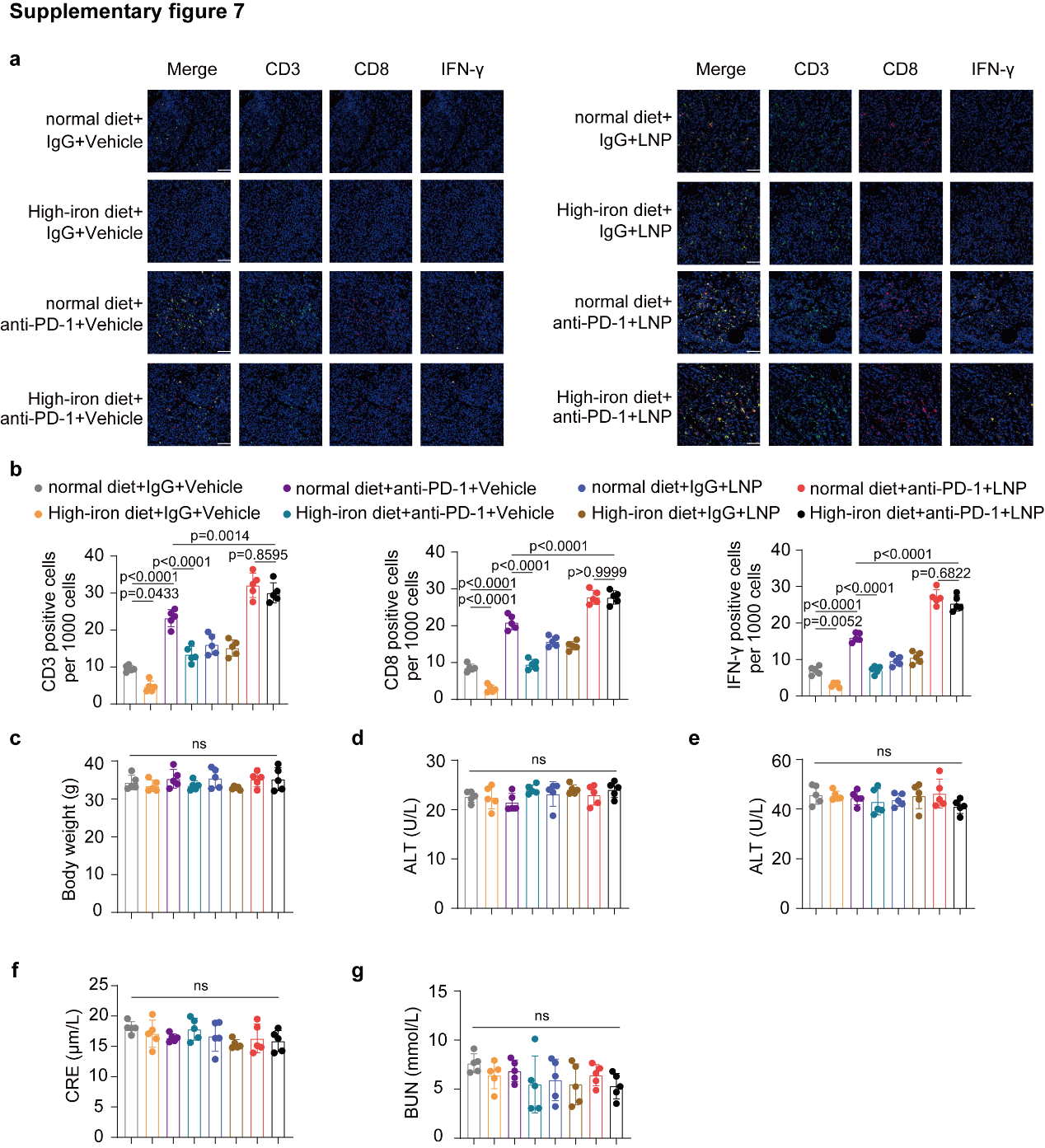


**Supplementary figure 7 (related to figure 7)**. **a-g**, BALB/c mice fed with HID or ND were subcutaneously injected with equal amounts of Renca cells, followed by treatment at the indicated doses on days 7, 10, 13, and 16 as shown in Fig. 7, with specific groups detailed in the figure legend. Mice were euthanized at appropriate time points and tumors were collected, with portions of tumor tissue subjected to multiplex immunofluorescence staining (a) for the indicated markers and statistical analysis (b) of each marker's proportion. Prior to euthanasia, mouse body weight (c), ALT (d), AST (e), CRE (f), and BUN (g) levels were measured. ns, not significant.

**Table S1. The shRNA and sgRNA sequences.**

| shZDHHC12 #1 | 5'- GATCGGTCAGTGGTTGCGGTCCATTCTCGAG AATGGACCGCAACCACTGACCTTTTTG -3' |
| --- | --- |
| shZDHHC12 #2 | 5'- GATCAGGAGGAGCTCAAAGAGGATTCTCGAG AATCCTCTTTGAGCTCCTCCTTTTTTG -3' |
| shFDX1 #1 | 5'- GATCGCAATCACTGATGAGGAGAATCTCGAG ATTCTCCTCATCAGTGATTGCTTTTTG -3' |
| shAFG3L2 #1 | 5'- GATCGGACGCTTTACCGATTTGTTTCTCGAG AAACAAATCGGTAAAGCGTCCTTTTTG -3' |
| shAFG3L2 #2 | 5'- GATCGGAAGGACTTTGTCAATAATTCTCGAG AATTATTGACAAAGTCCTTCCTTTTTG -3' |
| sgAFG3L2 | 5'- CTTCATCGATGAAATCGATGCGG -3' |
| sgAFG3L2 (mouse) | 5'- TTACCACGAAGCAGGCCATGCGG -3' |
| sgTRIM28 | 5'- AGTTCTTAGAGGATGCAGTGAGG-3' |

**Table S2. The primer sequences for RT-qPCR.**

| **Species** | **Gene** | **Forward (5’-3’)** | **Reverse (5’-3’)** |
| --- | --- | --- | --- |
| Human | β-actin | GCCTCGCCTTTGCCGAT | AGGTAGTCAGTCAGGTCCCG |
| Human | FDX1 | CAGCGGCCTGCTGAGG | ACTGTTATTTTATCTTCTGAGCTGC |
